# Mapping the global opinion space to explain anti-vaccine attraction

**DOI:** 10.1038/s41598-022-10069-3

**Published:** 2022-05-19

**Authors:** Dino Carpentras, Adrian Lüders, Michael Quayle

**Affiliations:** 1 Social Dynamics Lab, Department of Psychology, Centre for Social Issues Research, University of Limerick, Limerick, Ireland; 2 MACSI (Mathematics Applications Consortium for Science and Industry) Department of Mathematics and Statistics, University of Limerick, Limerick, Ireland; 3 Department of Psychology, School of Applied Human Sciences, University of KwaZulu-Natal, Scottsville, South Africa; 4 Lero, The SFI Research Centre for Software, University of Limerick, Castletroy, Limerick, Ireland

**Keywords:** Human behaviour, Health policy

## Abstract

Vaccines save millions of lives every year. They are recommended by experts, trusted by the majority of people, and promoted by expensive health campaigns. Even so, people with neutral attitudes are more persuaded by people holding anti-vaccine than pro-vaccine attitudes. Our analysis of vaccine-related attitudes in more than 140 countries makes sense of this paradox by including approaches from social influence. Specifically, we show that neutral people are positioned closer to anti- than to pro-vaccine people in the opinion space, and therefore more persuadable by them. We use dynamic social simulations seeded with vaccine survey data, to show how this effect results in a drift towards anti-vaccine opinions. Linking this analysis to data from two other multi-country datasets, we found that countries in which the pro-vaccine people are less associated to the neutrals (and so less able to influence them) exhibit lower vaccination rates and stronger increase in distrust. We conclude our paper by showing how taking social influence into account in vaccine-related policy-making can possibly reduce waves of distrust towards vaccination.

## Introduction

### The anti-vaccine paradox

Vaccines save millions of lives every year^1–3^. Despite their safety and efficacy, vaccine coverage has been decreasing in several countries leading to outbreaks of previously well-controlled diseases such as measles^4,5^. Consequently, in 2018 the world health organization has declared vaccine hesitancy a major threat for global health^3^.

Understanding how people may be persuaded towards anti-vaccine positions is therefore critically important^6^. We already know that people holding neutral opinions (hereafter: the neutrals) are more influenced by people holding anti- than pro-vaccine attitudes (hereafter: the anti-vaccine and pro-vaccine population)^7^. However, the striking influence of anti-vaccine population on the neutrals is puzzling since there would be many reasons to predict the opposite.

Indeed, governments are dedicating substantial resources to promoting confidence in vaccines (for example, Australia is spending $23.9 million to promote COVID-19 vaccines^8^). Furthermore, the number of people holding pro-vaccine attitudes is roughly 8 times bigger than the number of people holding anti-vaccine attitudes^9^. This includes a strong multi-sectoral and multidisciplinary consensus of experts^10^. Finally, people holding anti-vaccine attitudes are often viewed by mainstream society in a derogatory way^11^. Pro-vaccine positions are therefore more normative and higher status. Furthermore, to date, no special skill has been found that can explain the superior persuasive power of the anti-vaccine population in influencing the neutrals. For all these reasons, we would expect the neutrals to be more persuaded towards the pro-vaccine side and not the opposite.

In this article we solve this paradox by showing how the neutrals are, in terms of attitudes, closer to the anti than the pro-vaccine people. As we will discuss, this attitudinal closeness is directly related to the ability of influencing other people. Indeed, we will show both in simulations and in real data that the anti-vaccine population is more likely to grow in countries in which the pro-vaccine population shows greater attitudinal distance to the neutrals. Importantly, attitudinal distance also hold informational value for concrete behavior in that greater distances between the neutrals and the pro-vaccine population predict lower level of vaccination coverage.

### Social influence, similarity and distance

In recent years, there has been great interest in understanding and modelling social influence^12,13^. These models focus on how people are influenced by their peers, and they are commonly based on the principle that people are more influenced by those similar to them. This principle is supported by experimental evidence^14^ and well-established concepts and theories, such as homophily^15–17^, confirmation bias^18^ and social judgement theory^19^.

In models of social influence, similarity between people is sometimes represented as an overlap^20^ (e.g. number of attitudes in common) or, more commonly, as a distance^12,21^. Specifically, the closer two people are, the more similar their attitudes will be. For example, according to these models, people holding left-wing attitudes (in the left–right space of political attitudes) would be extremely far from people holding right-wing attitudes. However, they would be closer to a person holding neutral attitudes. Since distance is also connected to influence, this means that for a right-winger it will be much harder to persuade a left-winger than a neutral person.

This concept of distance can be applied not only to people, but also to attitudes^22^. Thus, the attitude “support gun control” would be distant from “oppose gun control” but closer to a neutral attitude. This space in which people or attitudes are closer or further depending on how similar they are is often referred as “opinion-space” or “attitude-space” ^12,23^. As we will see, this is a very powerful tool for visualizing and understanding why the neutrals are more influenced by the anti-vaccine and less by the pro-vaccine population.

### Attitude space and proximity

#### Visualizing and understanding social influence

While the main idea of social influence may appear intuitive for many people (i.e. the more similar two people are, the more they could exert reciprocal influence) the overall phenomenon can be rather complex to study. This is mostly due to the dynamic and non-linear nature of the process. Because of that, these models are often studied through specific computer simulations, called agent-based simulations^12^.

**Figure 1:**
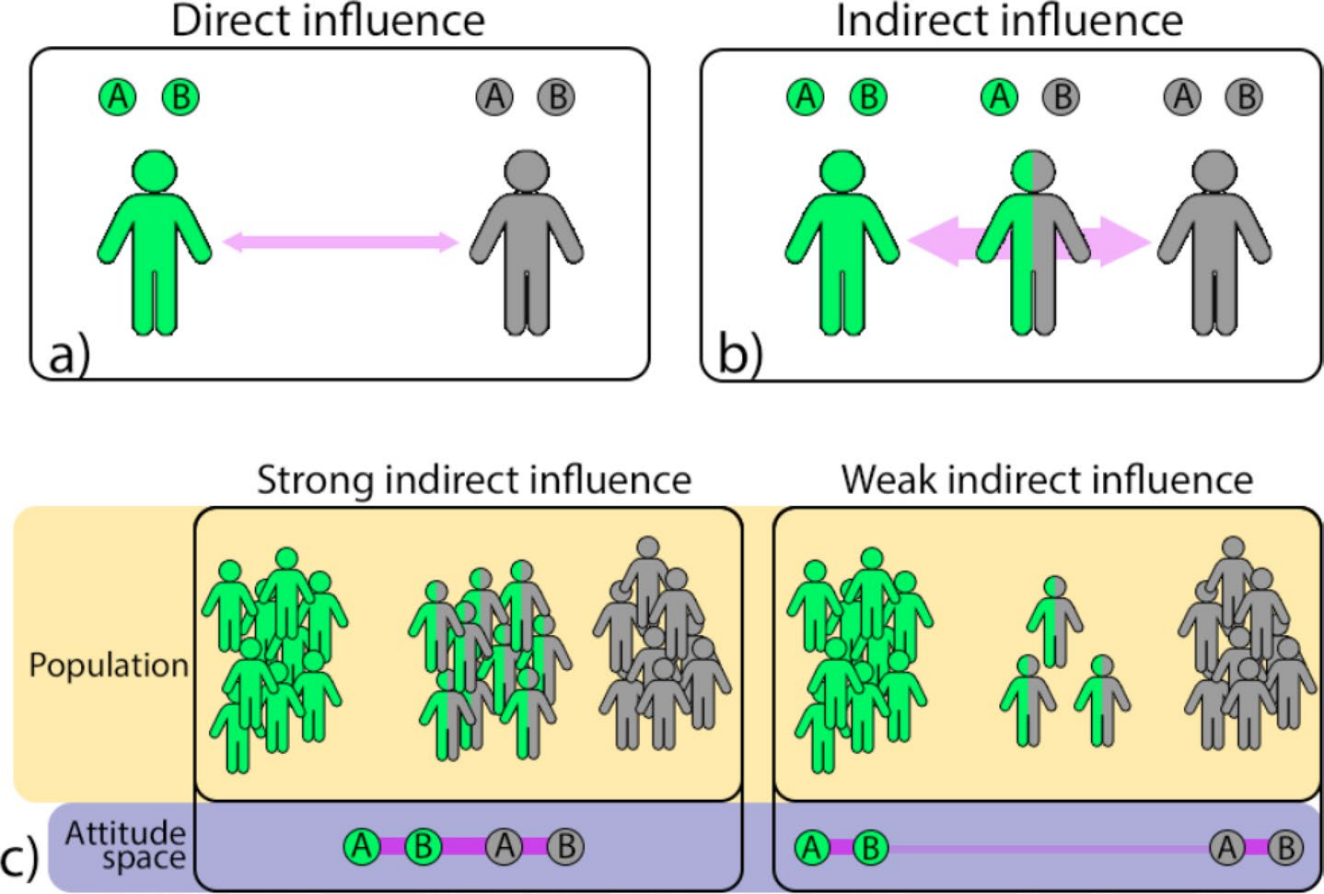
(**a**, **b**) Representation of social influence between the positive people (green) and the neutrals (grey). (**a**) A direct interaction does not exert much influence, as the two have little in common. However, (**b**) influence can be strengthened by the presence of people having something in common with both. (**c**) Similar representation in a population (orange area) and how this indirect social influence is represented in the attitude space (violet area).

While later we will carefully analyze the influence process through both agent-based simulations and real-world data, at this stage we want to provide a naïve, but intuitive, interpretation of the key phenomenon involved. For doing so, let us consider an example in which we have only two topics (e.g. immigration and gun control) labeled as A and B in Fig. 1. Also, for simplicity, let us consider only two levels: positive and neutral; visually represented as green and grey. Each person could only be in one of the following three states: positive on both A and B (represented as green people), neutral on both (grey people), positive on one topic and neutral on the other (represented as half-green and half-grey). Furthermore, suppose that the positive outnumber the neutral people, as is the case for vaccination, where people who trust vaccines outnumber the neutrals^9^.

Despite their number, the positive people may still struggle in influencing the neutrals as the two groups have little in common. A half-positive half-neutral person, instead, would be much closer to a neutral person, thus, being more able to influence her. Furthermore, half-positive half-neutral people are themselves influenced by the positive people. This means that the positive people, in order to influence the neutrals, do not just need a big population, but also enough people with an intermediate point of view to act as “intermediary” of this indirect social influence.

As we will see, the attitude space can be used to visualize indirect influence. In the next section we will also carefully explain how it can be obtained from real-world ﻿data, while here we just want to give an intuitive understanding of its working principle.

In the attitude space, two attitudes (represented as circles) appear closer and tightly connected if they are usually held together by people. For example, in both panels of Fig. 1c many people are positive about topics A and B (i.e. the fully green people). Because of that, the two green attitudes appear close to each other in the attitude space (violet area of the figure). On the contrary if two attitudes are rarely held together, they will appear far apart to each other and weakly connected. To put it simply, if two attitudes X and Y appear close to each other, we could read it as the fact that “people who think X usually also think Y”.

As we can see from the attitude space in Fig. 1c, if the green attitudes and the grey ones are tightly connected, it means that there are enough half-positive half-neutral people; therefore, indirect influence can occur. Instead, if their connection is weak or absent, it means that there is only a small number of people for carrying out indirect influence.

### Network-based analysis

To map the attitude-space for vaccine-related attitudes, we analyzed data from the Wellcome Global Monitor, containing data from 144 countries and 149,014 people ^9^. This is currently the most extensive dataset on vaccine-related attitudes. In this dataset, participants answered questions on several topics including trust in vaccines, trust in science and trust in public institutions. We selected all the questions on these topics which had four response options (Strongly Agree, Mildly agree, Mildly disagree and Strongly disagree) and the three questions on vaccines which also included a neutral option. Here we refer to each response option with the term “attitude”. For example, “Vaccines are effective: strongly agree” will be a different attitude from “Vaccines are effective: weakly agree”.

**Figure 2:**
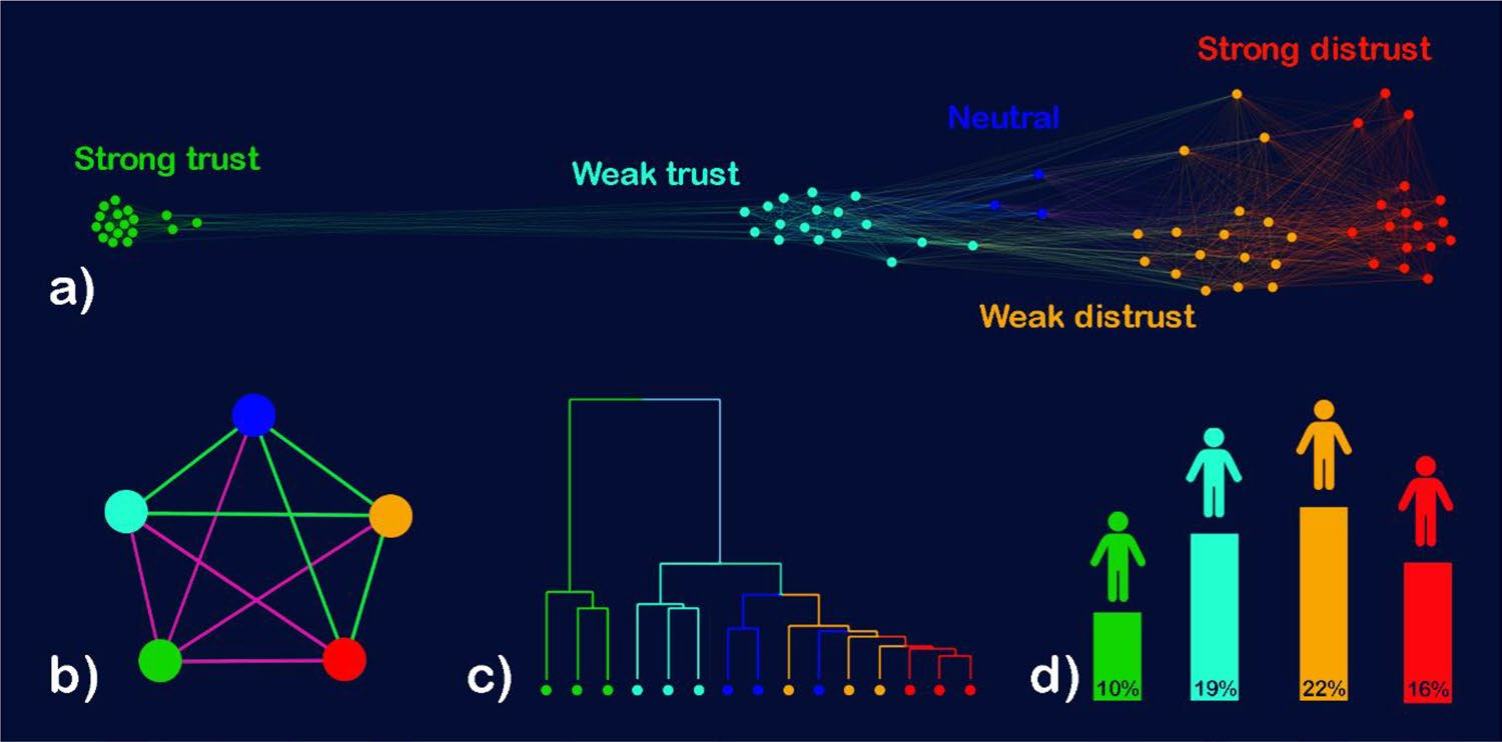
Isolation of the pro-vaccine attitudes. In all sub-figures green represents “Strong Trust,” pale blue “Weak Trust,” dark blue “Neutral,” orange “Weak Distrust,” and red “Strong Distrust”. (**a**) Opinion space obtained from the Wellcome Global Monitor. (**b**) Correlation between vaccine-related attitudes. Green edges are positive, violet ones are negative. (**c**) Hierarchical clustering of answers. (**d**) Probability of people from different groups (e.g. Strong Trust) to hold also neutral attitudes.

To estimate and visualize indirect influence in the attitude space we used a network-based method (see Fig. 2a). In this network every node is a response option, and the weight of the link is the correlation between two response options.

Specifically, we dummy-coded^24^ each attitude. This means that each response option for each question (e.g. “*Trust vaccines: Strongly agree*”) becomes a binary variable. Alternatively, this operation can be explained as replacing each item with 4 or 5 (depending on the number of levels) binary variables. For example, the item “*Trust vaccines”* would be replaced by 5 binary variables: “*Trust vaccines: Strongly agree”*, “*Trust vaccines: Mildly agree”*, etc. More details, as well as the full code, can be found in the Supplementary information.

For estimating the weight of the link between two binary variables (i.e. attitudes), we calculated the phi correlation coefficient. This value, for binary variables, coincides with the Pearson’s and Spearman’s correlation coefficient^25^. We followed this method because it does not assume any measurement properties beyond ordinality, which is important when using Likert-type items. Since attitudes belonging to the same item are mutually exclusive (i.e. if one’s value is 1 the other should be 0) we did not calculate correlation between them, as this did not contain any information about the attitude space.

To confirm that the network is not due to random fluctuations of the data, we calculated its p-value through bootstrapping^26^. In each bootstrap sample, we shuffled the attitude-response for each attitude across participants. Thus, preserving the number of ones and zeros in each column but changing their order. This method also preserved the constraint that each person may select only one answer per question. For each resample, we produced the network as described above and summed up the absolute value of the edges weight (i.e. the correlation between attitudes). This sum was used as test statistic, so for measuring how rare each result was. In the real data, we obtained a value of 101, while the maximum value after 10,000 bootstrap iterations was 64. This means that even after 10,000 random trials we were not able to produce a result as rare as the original one. This results in a value of p < 0.0001 meaning that such a network is very likely not produced just by statistical fluctuations and, therefore, meaningful.

The network produced in this way is graphically represented in Fig. 2a by using Gephi’s “Force atlas 2” algorithm^27^. In this specific representation we did not include negative links (i.e. we dropped them) because the visualization algorithm does not support negative edges. However, negative edges have been taken into account for all the other analyses.

In this representation, each node (i.e. circle) represents an attitude, while the links (i.e. lines) represent the (positive) correlation between them. The representation algorithm (i.e. Force atlas 2) is a force-directed method, meaning that links between nodes act as springs pulling them together. Therefore, nodes which are strongly correlated to each other will also appear close to each other. This is in agreement with the naïve explanation we gave in the previous section where if two attitudes appear close together it means that people who hold one tend also to hold the other attitude.

Finally, to improve readability of the graph, we color coded every node in accordance with their trust level (i.e. Strong trust as green, Mild trust as pale blue, Neutral as dark blue, etc.). This also allows us to double check the quality of the results. Indeed, we can see that nodes with the same color appear close to each other. This tells us, for example, that people who expressed mild trust on one item will also tend to express mild trust on another items, as we would expect. Similarly, we see absence of links between the extremes, meaning that people who expressed strong distrust on one topic will not be likely to express strong trust on another topic and vice versa.

Besides this initial confirmation, the network also shows us that the opinion space is not symmetric. Meaning for example, that the pattern that we observe for strong trust attitudes is different from the one of strong distrust.

This can be observed from the fact that the strong trust attitudes are tightly correlated together (visually represented by the compactness of the cluster). Thus, if a person holds one of these attitudes, they are very likely to also hold all the others. The situation is quite different for the other attitudes. For example, people holding a strong distrust attitude would be likely to also hold a weakly distrust attitude and vice versa. In the figure, this is shown by the fact that orange and red attitudes are close together.

Strikingly, in this attitude-space, the strong trust attitudes are almost isolated from the rest of the system; thus, leaving the neutral attitudes closer to strong distrust. As previously mentioned, closeness in the attitude-space here serves as a proxy for social influence. Therefore this isolation of the strong trust attitudes may explain the initial paradox of the lower influence of the pro-vaccine people on the neutrals.

Before moving forward, as the concept of opinion space is still new, we want to first confirm in the data the observed separation of the strongly pro-vaccine attitudes from the rest of the attitude system. Because of this, we will use three independent methods.

### Confirming the isolation of the pro-vaccine side

To verify the observed separation of the strongly pro-vaccine attitudes from the rest of the attitude system, we selected only the questions on vaccination and analyzed them using three independent methods: average correlation, hierarchical clustering and conditional probability. The first two methods analyze patterns of similarity between attitudes, while the last analyzes similarities between people.

In the first method, we grouped the 15 vaccine-related attitudes by their level to produce five sets. Specifically, the strongly pro-vaccine attitudes form one set, the weakly pro-vaccine attitudes form another set, and so on. Then, we calculated the average correlation between each pair of sets. A negative correlation (represented by a pink link) means that people who hold attitudes from one set are less likely to hold attitudes from the second set. A positive correlation (represented by a green link), instead, means the opposite. In Fig. 2b (as well as in c and d) we kept the previous color coding based on the attitude level. Figure 2b shows that the strongly pro-vaccine set is negatively correlated with all the others (average correlation − 0.16), resulting in an isolated set.

Hierarchical clustering^28^ (Fig. 2c) iteratively groups the most similar attitudes at each step. Also for this method (as well as for the next one), we selected only the 15 attitudes on vaccination. Notice that here the variables are not independent as we are not trying to cluster a population from some independent variables. Instead, we are observing how different variables are clustered together based on how people selected them^29^.

Initially, (i.e. at the bottom of the figure) all the 15 attitudes are in different groups (i.e. different vertical lines). These were the same 15 dummy coded binary variables used for the previous analysis. We performed hierarchical clustering using Ward’s method^28,30^ provided in Python’s SciPy package^31^. As we move to the top, the most similar ones become grouped together. This is represented visually as two vertical lines merging into one. The algorithm keeps merging attitudes until all the elements are in a single group. Before this final step, two groups are found: one obtained by merging only the three strongly pro-vaccine attitudes and another containing all the other attitudes. Therefore, also, this method confirms the initial analysis of the attitude-space showing isolation of the strongly pro-vaccine attitudes from the rest.

Our final method looks at the probability of two people having at least one attitude in common with a fully neutral person. To calculate conditional probability (Fig. 2d), we selected the people that had at least one strongly pro-vaccine attitude and then identified the proportion of this subgroup who had at least one neutral answer. This value (10%) represents the probability that a person holding at least one strongly pro-vaccine attitude also holds at least one neutral attitude. Or, equivalently, the probability that they will have an attitude in common with a fully neutral person; thus increasing their influence on them^20^. We repeated this process for all the other levels (i.e. weakly pro-vaccine, weakly anti-vaccine and strongly anti-vaccine). The neutral one was not included, as the result would trivially be 100%.

As shown in Fig. 2d), only 10% of people who hold least one strongly pro-vaccine attitude also hold a neutral attitude. However, 16% of people with at least one strongly anti-vaccine attitude also hold a neutral attitude. Thus, this method also confirms that people holding strongly pro-vaccine attitudes are further from the neutrals than people holding strongly anti-vaccine attitudes.

We have now confirmed that the neutral position is closer to the anti-vaccine than to the pro-vaccine position both in terms of people and attitudes. As proximity in the attitude-space will generally result in stronger influence, this is one clear reason that neutrals are more likely to be persuaded by anti- than pro-vaccine population. Next, we will further explore this phenomenon with agent-based simulations.

Hereafter, due to the frequent use of this concept, we will refer to “the distance between the strongly pro-vaccine attitudes and the rest of the attitude system” with the term “attitude-isolation.” We calculate this value as the average correlation between the three strongly pro-vaccine attitudes and the 12 remaining ones. We then multiplied this value by − 1 to make it consistent with the concept of “isolation.” Indeed, as this values increases, the strongly pro-vaccine attitudes become more isolated from the rest of the system (as shown in Fig. 2a).

### Simulations of social influence

As people are more influenced from those who are already similar to them^12–14,20–22^, we expect the pro-vaccine population to be less influential when their attitudes are more isolated. Consequently, in these systems, the neutrals would be more influenced by people holding anti-vaccine attitudes.

While we already explained this idea in a naïve way, the complexity of social influence is rigorously studied through agent-based simulations. Therefore, to test the effect of attitude-isolation, we selected five different agent-based models of social influence. Specifically, we selected the Deffuant model^21^, three variations of the Hegselman–Krause (HK) model^32,33^, and Axelrod’s model of cultural diffusion^20^. These models are three extremely popular models in the social influence approach^12,13^. The two variations of the Hegselmann–Krause model^32,33^, instead, are more exotic, but we decided to use them to verify that even using non-linear averaging would not affect the model’s predictions. Indeed, while one model uses arithmetic mean (labeled as HK in Table 1), one variant uses the geometric mean (HK-G) and another the harmonic mean (HK-H).

**Table 1:** Summarizes the correlation coefficient found for each model and for each threshold value.

Model	Correlation threshold = 3	Correlation threshold = 6	Correlation threshold = 9	Correlation no threshold
Deffuant	− 0.42****	− 0.33****	− 0.29****	–
HK	− 0.34****	− 0.27****	− 0.46****	–
HKG	− 0.38****	− 0.36****	− 0.47****	–
HKH	− 0.43****	− 0.40****	− 0.50****	–
Axelrod	–	–	–	− 0.56****

The Axelrod model appears someway separated from the others as it does not use any threshold parameter. In all cases we observed a correlation with a strongly significant p-value (p ≪ 0.0001). Thus, these models predict that countries with higher attitude-isolation of the strongly pro-vaccine attitudes will also have higher increase of the anti-vaccine population. This has been then confirmed in the data analysis.

Since they are all models of social influence, they assume that people are more influenced by those who are similar to them. The main difference between models lay in how the influence process has been formalized. Discussing all the models in detail would go well beyond the scope of this article. Instead, we will outline the properties which are needed to understand the seeding process and the output data.

The Deffuant and Hegselmann–Krause models represent attitudes as a single number on one axis. Thus, the pro-vaccine opinions would be on one extreme and anti-vaccine ones would be on the opposite extreme of the same axis. We therefore converted the three answers on vaccine attitudes into a single number. Following psychometric convention, we coded responses numerically [Strongly disagree = 1, Weakly disagree = 2, Neutral = 3, Weakly agree = 4, Strongly agree = 5] and summed them^34^. Thus, a person who selected all three the strong trust answers will have a score of 3*5 = 15, while a person who selected all the strong distrust will have a score of 3*1 = 3. Cronbach alpha for this scale was 0.81. This coefficient is usually used to measure the quality of a psychological scale^34^. A value of 0.81 indicates good scale quality, therefore justifying the process of summing them together^35^.

The Deffuant and Hegselmann–Krause models depend also on a threshold parameter. To show that this does not influence our main claim, we tested each model using three different threshold values: 3, 6 and 9.

Since the results of these models are stochastic and produce slightly different results each time, each model was run 1000 times. For each run we used a bootstrap sampling method. We randomly chose one country and then, from the data of that country, randomly selected 1000 participants to initialize the agents. Since it is a bootstrap method, eventually, the same person may be selected more than once, thus the method works even for countries having less than 1000 respondents in the dataset. In order to track the skeptical population, we identified all the neutral and negative people in the sample (i.e. people with summed score of the three items ≤ 3*3 = 9) and recorded their average score at the start and end of the simulation. Notice that, due to social influence, some of them may have moved towards more positive values by the end of the simulation, so the final average of the initially skeptics may eventually be above 9 after running the model. This means that we did not artificially impose any condition on this population and that their dynamics only depends on the model’s properties and the initial data.

The Axelrod model does not use distance, but instead represents people’s opinions as different topics and levels (in standard literature called *features* and *traits*). These two parameters are easily converted into the surveys’ questions and answers. The model does not require to represent opinion as a single number, however, to measure the position of the skeptics, we also checked the score of each person. In this way we could identify skeptics using the same method as before (i.e. score ≤ 9), making the results of this model comparable with the previous ones. Also the final score of the skeptics has been obtained in the same way.

For each model’s run, we stored the final average opinion of the skeptics and the attitude-isolation parameter of the simulated country. Then, we calculate the correlation between these two variables. The results are shown in Table 1.

As we can see from the table, all five models showed a strongly significant correlation between attitude-isolation and lower trust in vaccines (all r between − 0.27 and − 0.56; all p-values smaller than 0.0001). Meaning that countries with stronger isolation of the pro-vaccine attitudes are more likely to have stronger anti-vaccine attitudes at the end of the simulation. This confirms that, in theory, isolation increases the influence of the anti-vaccine population on the neutrals. In the next section, we will confirm this finding also in real-world data.

### Predictions in real-world data

A unique feature of models of social influence is that they are dynamic, while many other models usually describe static relationships. For example, previous studies showed how trust correlates to vaccine-related behavior^36^. However, both trust and behavior data are from the same year, meaning that people are more likely to vaccinate their children as long as they trust vaccines.

Instead, the effect of attitude-isolation on behavior becomes manifest only after some time, as people need to interact in order to influence each other. Thus, attitude-isolation is expected to alter vaccine-related behavior not immediately, but in the following years; thus, predicting the future state of the system.

To confirm this idea, we analyzed three different international datasets. The first one is the Global Monitor 2018^9^. As previously done, we used this dataset to measure the attitude-isolation parameter in each country. The second dataset is the Global Health Observatory data repository of the World Health Organization^37^. This provides immunization rates for the second shot of the Measles Vaccine in 2018 and 2019 covering 176 countries. We obtained the increment of vaccination coverage in each country from 2018 to 2019 by subtracting the values for 2018 from 2019. Correlating this value with the attitude-isolation parameter calculated for each country that also appears in the Wellcome Global Monitor (N = 108) we obtain a correlation of r = − 0.31; p = 0.001. Therefore, telling us that countries with higher attitude-isolation are more likely to see a decrease in their vaccination coverage in the following year.

To make sure that this is not simply due to the effect of wealth or trust in vaccination (which have been shown to be the most important effects for estimating vaccination coverage^36^), we also calculated the partial correlation after removing these factors. Wealth was coded using the values provided for each country in the Wellcome Global Monitor. Country-level trust in vaccines was calculated using the method designed by the Vaccine Confidence Project^36^, where trust was estimated as proportion of people providing strongly positive answers to the vaccination questions. Controlling for these variables, the partial correlation between attitude-isolation and vaccine-uptake in the following years was still r = − 0.26; p < 0.01.

The third dataset comes from the already mentioned Vaccine Confidence Project^36^ which, for each country, reports the fraction of people responding strongly negatively to questions on trust in vaccines. This value has been used also in previous studies for quantifying the size of the anti-vaccine population^36^. For some countries two datapoints were available for 2018, in these cases, the earliest one was chosen. Unfortunately, data for both 2018 and 2019 were available for only 43 countries, strongly reducing the sample size and data significance from the previous analyses. However, the obtained correlations were still significant. Indeed, we found that the correlation between the increment in distrust and the attitude-isolation parameter was 0.32; p < 0.05. This tells us that countries with higher attitude-isolation are also more likely to see an increase of vaccine distrust in the following year.

While the obtained correlations may seem relatively small, it should be considered that they refer to a complex social phenomenon that is influenced by a large number of different factors (e.g. politics, social events, etc.). For example, despite its great importance for this phenomenon, trust in vaccines’ safety has been identified as having a correlation of 0.28 with children vaccination^36^.

In this section, we showed how the attitude-isolation parameter can inform us about future levels of trust and vaccination uptake in one country. In the next section, we discuss how this could (and should) be considered in policy making.

### Relevance for policy intervention

Policy-makers addressing vaccine hesitancy are well-aware of the importance of parameters such as trust^36^. Because of that, massive funding is spent in promoting trust in vaccination^8^. However, increasing people’s trust in vaccination without considering the attitude-space may have unintended effects by increasing attitude-isolation and thus, reducing the influence of the pro-vaccine population. Furthermore, while trust appears to have an immediate effect, attitude-isolation’s effect is dynamic, thus appearing only in the following years. This means that after an intervention, in the short run, we may observe an immediate increase of trust in vaccination, but the neutrals may still be more influenced by the anti-vaccine population. Hence, in the following years, we will observe downstream increases in distrust.

Even though this paper is not primarily about policy-making, we want to show the importance of developing policies that are sensitive to the problem of attitude-isolation. To do so, we simulated two similar fictitious policies aimed at increasing trust in vaccines.

The population parameters are obtained from the Wellcome Global Monitor 2018^9^ and refer to the three vaccine-related questions. When a person is targeted by our simulated policy, she increments her trust by 1 point in one of the three attitudes. For example, if a person initially moderately agrees that vaccines are important, after being targeted she will strongly agree. Attitudes are selected only if they are not already at maximum (i.e. Strong trust). People who already hold all three strongly pro-vaccine attitudes will not be targeted. Both policies successfully target 5% of the population, thus producing the same result in terms of immediate increase of trust. After running the simulated intervention, we checked again the attitude-isolation parameter to measure how it has been affected by the intervention.

Policy 1 influences only people who are almost sure about vaccines but still not completely. This could be a natural choice for policy makers as these people should be the easiest to reach and convince^38,39^, boosting vaccine support with minimal effort. The effect of policy 1 is to immediately increase the level of trust in vaccination. However, this policy also reduces the number of people holding both strongly and weakly pro-vaccines attitudes as it pushes them to hold only the strongly positive. In our naïve example, this would be equivalent to remove the half-and-half people by making them all green. This results in a weakening of the link between strongly and weakly pro-vaccine attitudes. As result, the attitude-isolation level is increased by 31% therefore weakening the influence between the pro-vaccine people and the neutrals.

Policy 2, instead, is designed not only to immediately increase trust in vaccines, but also to decrease the attitude-isolation parameter. To do so, it targets people who have only weakly pro-vaccine attitudes. By increasing the trust value of some of their attitudes, we produce more people holding both strongly and weakly pro-vaccine attitudes. Indeed, this policy decreases attitude-isolation levels by 52%, thus strongly decreasing possible new waves of distrust.

## Discussion

Previous studies have focused on how to make vaccines or institutions appear more trustworthy to public’s eyes^1,8,36^. However, this top-down approach neglects the fact that people interact, thus influencing each other’s attitudes. This means that when analyzing the problem of vaccine-hesitancy, we should not limit our analysis to individual measures of trust. Instead, we should also carefully study how trust can spread through the social system. This can be used to avoid future waves of distrust, as well as finding the optimal strategies for a cohesive and healthy societies.

In this work, we showed how studying the attitude space and, specifically, the attitude-isolation parameter is a powerful tool for this goal. We initially provided a naïve and intuitive explanation for this phenomenon and then validated it on more rigorous theory via simulations and on empirical data.

This tells us that the attitude-isolation parameter can be used for analyzing which countries are at risk of increasing the anti-vaccine sentiment in the near future. Furthermore, that policy makers should consider the possible impact of their policy on attitude-isolation as this may result in backfiring and, therefore, producing unanticipated consequences such as new waves of distrust.

Despite the relevance of this work, we want to stress that we did not take into account parameters such as stubbornness or other personal traits. While models of social influence with these characteristics exists^40^, we preferred to not include them in this first study as they would increase its complexity and require more assumptions (e.g. specifying how stubbornness is distributed in the population). However, future studies should also consider that different parts of the population may behave differently. Specifically, multiple studies have highlighted how neutrals and anti-vaccine people may have very different reasons for their behavior and respond differently to the same stimuli^41^.

In this study, we applied social influence and the concept of attitude-isolation to the problem of vaccine hesitancy revealing a core dimension that has not been visible until now. In the future, this approach could be applied to other problems, such as climate change and the spread of conspiracy theories^7,42^, thus finding new ways to address some of the most pressing social issues.

## Supplementary Information

Supplementary Information.

## Data Availability

Code can be found at: https://github.com/just-a-normal-dino/isolation_pro_vax_full
