# Supplementary material for: Mapping the global opinion space to explain anti-vaccine attraction
Source: Sci Rep. 2022 May 19;12:6188. doi: 10.1038/s41598-022-10069-3 (PMC9120185; doi:10.1038/s41598-022-10069-3)
Supplement: Supplementary file 1 — Supplementary Information. [file 41598_2022_10069_MOESM1_ESM.pdf]

Contents

I Overview of the methods 4

II Pseudocodes 6

1 Dummy coding 6
1.1 General information 6
1.2 Pseudocode 6

2 Making the correlation network 8
2.1 General information 8
2.2 Pseudocode 8

3 Social influence models 9
3.1 General information 9
3.2 Pseudocode 9

III Clean and dummy code the WGM dataset 11

4 Import the packages 11

5 Import the raw data 11

6 Clean the dictionary 13

7 Define functions acting on the dictionary 16
7.0.1 Translate questions 16
7.0.2 Tranlsate answers 18

8 Clean the labels in the database 20
8.0.1 Make the numeric version of the database 20
8.0.2 Make the version with labels of the database 21

9 Boolean version of the database (aka dummy coded) 23

10 End of cleaning 24

IV Figure 1 and relative analysis 25

11 Import the packages to use 25

12 List of attitudes 25

13 Functions 32

14 Making figure 1a 37

14.0.1 Calculate the p-value 40

15 Make figure 1b **42**
15.0.1 Make the second graph 42
15.0.2 Additional visualization 44

16 Fig 1c **45**

17 Fig 1d **47**

V Models of social influence **50**

18 Import packages **50**

19 Functions & relative classes **50**
19.0.1 Special functions 50
19.0.2 Functions for labelling 52
19.0.3 Functions for the Network 52
19.0.4 Functions for Initializing opinions 53
19.0.5 Selection rules 55
19.0.6 Update rules 57
19.0.7 Observables 61

20 Functions for running and ticking **62**
20.0.1 Ticks 62
20.0.2 Run 62

21 Main class **63**

22 Functions for dealing with the data **63**

23 Lists **65**

24 Initialize the data **66**

25 Calculate the isolation parameter for each country **68**

26 Run the models **69**
26.0.1 Deffuant 69
26.0.2 HK (standard mean) 72
26.0.3 HK with geometric mean 75
26.1 HK with H-mean 78
26.1.1 Axelrod model 81

VI Correlations and predictions **85**

27 Import libraries **85**

28 List of attitudes **85**

29 Functions **90**

30 Import the datasets **93**
30.0.1 Dataset on vaccination coverage 93

31 Dataset on attitudes towards vaccination **97**

32 Test the correlations **98**
32.0.1 Prediction in vaccination coverage 98
32.0.2 Test prediction in Trust 101

33 Test predictions from confidence **104**

VII Simulating policies **106**

34 Import packages **106**

35 Functions **106**

36 Lists **108**

37 Calculate the initial isolation value **109**

38 Test policy **110**
38.0.1 Bad method 110
38.0.2 Intervention tailored at decreasing isolation 111

Supplementary methods

Part I

Overview of the methods

This document contains the additional materials to reproduce the results and improve transparency. Specifically, Part II includes the pseudocodes of the most important procedures. Instead, parts III to VII are the (commented and detailed) python codes used to produce the results.

Specifically:

- Part III explains how to clean and dummy code the original dataset from the Wellcome global monitor.
- Section IV shows how to obtain figure 1 (and relative sub-figures) as well as the relative analysis of the data.
- In Part V we use models of social influence to show that the isolation parameter has a detrimental effect on the system
- In Part VI we analyze another two datasets to show that the isolation parameter correlates with lower vaccination rate and increase in anti-vaccine attitudes
- Finally, in Part VII we simulate two different policies for promoting trust in vaccines. For each one we also look at the impact on the isolation parameter. Thus showing that different policies may have strongly different effects.

We wrote the code using Jupiter notebook, so it is possible to inspect it, analyze it and run it step by step. Specifically, Jupiter notebook allows to include text and figures which could not be included in standard code. In this way it should be way simpler for readers to analyze and reproduce the results from the codes.

All the codes and the package "wgm2018_package" are available at:

https://anonymous.4open.science/r/isolation_pro_vax_full-3FD4

The used databases are:

- Wellcome Global Monitor, available at: https://wellcome.org/reports/wellcome-global-monitor/2018

- The data repository of the World Health organization, available at https://apps.who.int/gho/data/node.main.A824?lang=en
- The repository of the Vaccine Confidence Project, available at: https://www.thelancet.com/journals/lancet/article/PIIS0140-6736(20)31558-0/

Part II

Pseudocodes

1 Dummy coding

1.1 General information

The first pseudo-code is the one transforming a standard database into a dummy coded one. The main difference between the two is that in the first one each column represents a question (i.e. an item), while in the second case, each column is a combination of the question with a single answer (i.e. an item-response).

For example, suppose there is only one question in the dataset: "Are vaccines effective?" Suppose also that the possible answers are: 1=Agree, 2=Neutral, 3=Disagree. Then the first dataset will contain only one column. In each of its raws we will find one number ranging between 1 and 3 (depending on the person's answer).

In the second case, instead, we will have three columns: "vaccines-effective:agree," "vaccines-effective:neutral," and "vaccines-effective:disagree." For each raw, all the columns will be zero, except the one of the selected answer. For example, if the person selected the answer "neutral" the only non-zero entry would be "vaccines-effective:neutral."

The figure below shows the transformation between the two datasets.

Original dataset

q1
1
3
2
2
1
3
3
2
1
2
3

Dummy coded dataset

q1:1	q1:2	q1:3
1	0	0
0	0	1
0	1	0
0	1	0
1	0	0
0	0	1
0	0	1
0	1	0
1	0	0
0	1	0
0	0	1

1.2 Pseudocode

Pseudocode 1: Dummy coding of a dataframe

```
dummy_df ← empty dataframe
for column in dataframe_original
    unique_values ← uniqueValues(column)
    for val in unique_values
        column_temp ← column == val
        append_col_to_df(dummy_df, column_temp)
```

The explanation of the variables and functions is the following:

- *dummy_df* is the dataframe we want to obtain. It is initialized as an empty one
- *dataframe_original* is the initial dataframe
- uniqueValues() is a function which provides as output only the unique values of a column. E.g. the output of uniqueValues([1,1,5,100, 100, 5]) would be [1,5,100]
- the command "a == b" produces a new column whose entries are either True or False. The entry would be true where the two corresponding entries in a and b would be equal. The entry would be False otherwise
- append_col_to_df() is a function which adds a column to a dataframe

2 Making the correlation network

2.1 General information

Once we have the dummy coded dataframe, we can generate the (weighted) network just by calculating the correlation between each couple of columns. In the network, each column would be represented as a node and the weight of the edge between them would the the correlation of the corresponding columns.

2.2 Pseudocode

Pseudocode 2: Making the correlation network

```
dummy_graph ← empty graph
for column1 in dummy_df
    for column2 in dummy_df
    if not belong_to_same_question(column1, column2)
        corr ← correlation(column1, column2)
        add_edge_to_graph(graph= dummy_graph, node1=column1, node2=column2,
                edge_weight=corr)
```

The explanation of the variables and functions is the following:

- *dummy_graph* is the graph we want to obtain. It is initialized as empty.
- not belong_to_same_question() is a function which outputs True if the two columns belong to the same question (remember that each column here is a specific answer of a question).
- add_edge_to_graph() is a function which adds the weighted edge between two nodes. If the two nodes are not already in the graph it will add them too.

3 Social influence models

3.1 General information

The following pseudocode refers to the general structure of models of social influence (e.g. Deffuant model). Even if each model is different, they still have a similar general structure. Specifically, they have an "initialization phase" where the attitudes of the agents are initialized according to a specific distribution. A "selection rule" which determines how agents are selected and under which conditions they can interact (e.g. if their difference of opinions are below a certain threshold). An "update rule" which determines how agents change their attitudes after the interaction.

3.2 Pseudocode

Pseudocode 3: running social influence models

```
initialize_agents_opinions_from_data()
for i=1 to N_rep
    [a,b] ← select_agents()
    if agents_can_interact(a,b)
        [a,b] ←update_agents_opinions(a,b)
```

The explanation of the variables and functions is the following:

- initialize_agents_opinions_from_data() is a function which initialize the attitudes of the agents to reflect the data from the Wellcome Global Monitor
- *N_rep* is the number of iterations of the model
- select_agents() is a function which selects the agents according to the selection rule (more information below).
- agents_can_interact(a,b) is a function which checks if the two agents can interact (according to the interaction rule of the model). If they can, the function will update a True value.
- update_agents_opinions(a,b) is a function which updates the attitudes of agents a and b accordingly to the update rule

Regarding the interaction rule

- both in the Deffuant and in the Hegselmann-Krause model agents can interact if their opinion difference is below the threshold.
- in the Hegselmann-Krause models you will randomly select a. b, instead, would be the set of all the agents whose opinion is similar to a's one. Meaning that their difference of opinions is below the threshold

Regarding the selection rule:

- both in the Deffuant and in the Hegselmann-Krause model you will just select two random agents a and b
- Instead, for the Axelrod model, the interaction will happen with a probability which is proportional to the number of shared attitudes between the two agents.

Regarding the update rule:

- In the Deffuant model both agents will end up with an opinion which is the average of the two.
- The Hegselmann-Krause model works really similarly, except that the mean is calculated not between two agents, but in a group of agents (a and b together). Also, different models will use a different mean (e.g. geometric or harmonic mean)
- For the Axelrod model if two agents can interact, agent a copies one attitude from agent b. a can only copy an attitude that she does not already have. If they are already equal, nothing happens.

Part III

Clean and dummy code the WGM dataset

In this notebook we will import the raw data from the WGM, clean them and dummy-code them in order to make them compatible with the future analysis we will run.

[ ]:

4 Import the packages

[3]:

```
import pandas as pd
import numpy as np
import scipy.stats as stt
import networkx as nx
import matplotlib.pyplot as plt
```

[ ]:

5 Import the raw data

Main data:

`wgm_raw` the full database

`wgm_dic` a dictionary of what the database means -> the important columns are the `code`, `long question` and `short question`

Note: wgm_dic is not a dictionary data type, but a dataframe. This has been done as we need to convert between 3 different types of dataframe we will deal with:

- boolean (i.e. dummy coded)
- labels (i.e. very entry is
- numeric

The file `wgm2018.xlsx` is the raw file provided by the Wellcome Global Monitor: https://wellcome.org/reports/wellcome-global-monitor/2018

Instead, the `wgm2018_data_dic_mod.xlsx` is a file made by us to rename the questions and the answers in a more compact way for when dummy coding. You can find it here: https://github.com/just-a-normal-dino/wgm18_dic

[4]:

```
# Import the raw data
wgm_raw = pd.read_excel('wgm2018.xlsx', sheet_name=1)
wgm_dic = pd.read_excel('wgm2018_data_dic_mod.xlsx')
wgm = wgm_raw.copy()
```

Display the raw data

[5]:
```
# wgm_raw.info()
wgm.head()
```

[5]:
```
   WP5       wgt         PROJWT FIELD_DATE  YEAR_CALENDAR  Q1  Q2  Q3  Q4  \
0    1  0.652821  171769.597742 2018-01-08           2018   3   2   1   2
1    1  0.695706  183053.484155 2018-01-08           2018   2   2   1   2
2    1  0.523829  137829.328857 2018-01-08           2018   2   2   1  98
3    1  0.764442  201139.215039 2018-01-08           2018   2   1   1   2
4    1  3.327946  875645.512738 2018-01-08           2018   2   1   1   2

   Q5A  ... Age AgeCategories  Gender  Education  Urban_Rural  \
0    2  ...  72             3       2          3            1
1    1  ...  72             3       1          2            2
2    1  ...  85             3       1          2            1
3    1  ...  54             3       1          3            2
4    1  ...  20             1       1          2            2

   Household_Income  Regions_Report  Subjective_Income  WBI  EMP_2010
0                 3               7                  2    4         6
1                 3               7                  1    4         6
2                 2               7                  3    4         6
3                 5               7                  1    4         1
4                 2               7                  1    4         6

[5 rows x 60 columns]
```

Display the dictionary

[7]:
```
wgm_dic.head()
```

[7]:
```
            Code                                     Long question  \
0            WP5                                           Country
1            wgt  National weight, for analysis at the country l...
2         PROJWT  Population weight (included factor to project ...
3     FIELD_DATE                               Study Completion Date
4  YEAR_CALENDAR                                     Year of survey

     Short question  Trust in science value  \
0           Country                       0
1        Nat weight                       0
2        Pop weight                       0
3   Completion Date                       0
4       Survey Year                       0

                                         Ans dic  \
0  1=United States, 2=Egypt, 3=Morocco, 4=Lebanon...
1                          Scale (value of weight)
```

```
2                             Scale (value of weight)
3                                                Date
4                                                Year

                                                Notes
0                                                 NaN
1  Use this weight for analysis at the country level
2  Use this weight for analysis which pools toget...
3                                                 NaN
4                                                 NaN
```

[ ]:

[ ]:

6 Clean the dictionary

Drop the notes column

[8]:
```python
# Note: if you'll run this cell twice, you'll get an error as it cannot delete␣
↪it twice
wgm_dic.drop(columns="Notes", inplace=True)
wgm_dic.head()
```

[8]:
```
            Code                                      Long question  \
0            WP5                                            Country
1            wgt  National weight, for analysis at the country l...
2         PROJWT  Population weight (included factor to project ...
3     FIELD_DATE                               Study Completion Date
4  YEAR_CALENDAR                                     Year of survey

    Short question  Trust in science value  \
0          Country                       0
1       Nat weight                       0
2       Pop weight                       0
3  Completion Date                       0
4      Survey Year                       0

                                              Ans dic
0  1=United States, 2=Egypt, 3=Morocco, 4=Lebanon...
1                             Scale (value of weight)
2                             Scale (value of weight)
3                                                Date
4                                                Year
```

Make the code column as the index of the dictionary (and duplicate it so I can easily access it as a column)

[9]:
```
wgm_dic["Code_i"] = wgm_dic["Code"]
wgm_dic.set_index("Code_i",inplace=True)
wgm_dic.head()
```

[9]:
```
                      Code  \
Code_i
WP5                    WP5
wgt                    wgt
PROJWT              PROJWT
FIELD_DATE      FIELD_DATE
YEAR_CALENDAR  YEAR_CALENDAR

                                                   Long question  \
Code_i
WP5                                                      Country
wgt            National weight, for analysis at the country l...
PROJWT         Population weight (included factor to project ...
FIELD_DATE                                 Study Completion Date
YEAR_CALENDAR                                     Year of survey

                Short question  Trust in science value  \
Code_i
WP5                    Country                       0
wgt                 Nat weight                       0
PROJWT              Pop weight                       0
FIELD_DATE     Completion Date                       0
YEAR_CALENDAR      Survey Year                       0

                                                         Ans dic
Code_i
WP5            1=United States, 2=Egypt, 3=Morocco, 4=Lebanon...
wgt                                  Scale (value of weight)
PROJWT                               Scale (value of weight)
FIELD_DATE                                              Date
YEAR_CALENDAR                                           Year
```

Add a new columns which tells you if the value is a cathegory or not (`Categorical Ans`). This would be true if the answers are categorical (aka "nominal"). And it would be false for continuous numeric variables such as age.

[11]:
```
ans_col = wgm_dic["Ans dic"]
is_category = ans_col.apply(lambda el : "=" in el) # Almost all categorical␣
 ↪variables have a dictionary in the form of "ans x = y"
wgm_dic["Categorical Ans"] = is_category
wgm_dic.loc[["Age"],["Categorical Ans"]] = False # Manually removing Age
```

```python
wgm_dic.head()
# print(wgm_dic.loc[is_category, ["Ans dic"]])
# print(wgm_dic.loc[wgm_dic["Categorical Ans"] == False, ["Ans dic"]])
```

[11]:
```
                      Code  \
Code_i
WP5                    WP5
wgt                    wgt
PROJWT              PROJWT
FIELD_DATE      FIELD_DATE
YEAR_CALENDAR  YEAR_CALENDAR

                                                  Long question  \
Code_i
WP5                                                     Country
wgt            National weight, for analysis at the country l...
PROJWT         Population weight (included factor to project ...
FIELD_DATE                                Study Completion Date
YEAR_CALENDAR                                    Year of survey

                Short question  Trust in science value  \
Code_i
WP5                    Country                       0
wgt                 Nat weight                       0
PROJWT              Pop weight                       0
FIELD_DATE     Completion Date                       0
YEAR_CALENDAR      Survey Year                       0

                                                        Ans dic  \
Code_i
WP5            1=United States, 2=Egypt, 3=Morocco, 4=Lebanon...
wgt                                       Scale (value of weight)
PROJWT                                    Scale (value of weight)
FIELD_DATE                                                   Date
YEAR_CALENDAR                                                Year

               Categorical Ans
Code_i
WP5                       True
wgt                      False
PROJWT                   False
FIELD_DATE               False
YEAR_CALENDAR            False
```

[ ]:

7 Define functions acting on the dictionary

As we will have three different dataframes in three different format (boolean, numeric and labels) here we define several functions to "translate" questions or answers from one dataframe to the others

[ ]:

```

```

Check if an element is in the series

[17]:

```
def is_in(series,element):
    #Checks if the element is in the series. If so, it also returns the index of␣
 ↪where it is found
    try:
        ind = series[series == element].index[0]
        out = [True, ind]
    except:
        out = [False, None]
    return out
```

[ ]:

```

```

7.0.1 Translate questions

Find the index of a question (in format string) from the dictionary (`wgm_dic`)

[18]:

```
def find_question_index(questions, in_format="Auto", out_format="Short"):
    # the question should be a string

    codes = wgm_dic["Code"]
    long = wgm_dic["Long question"]
    short = wgm_dic["Short question"]

    if type(questions) == type('abc'): # if it's a string

        isincode = is_in(codes,questions)
        isinlong = is_in(long,questions)
        isinshort = is_in(short,questions)

        if in_format == "Auto":
            if isincode[0]: # if it's a code
                ind = isincode[1]
            elif isinlong[0]: # if it's a long
                ind = isinlong[1]
            elif isinshort[0]: # if it's a short
                ind = isinshort[1]
            else:
                raise Exception("Question not found in any type!")
```

```
        elif in_format == "Code":
            if isincode[0]: # if it's a code
                ind = isincode[1]
            else:
                raise Exception("Question not found in the specified type!")

        elif in_format=="Short":
            if isinshort[0]: # if it's a code
                ind = isinshort[1]
            else:
                raise Exception("Question not found in the specified type!")

        elif in_format=="Long":
            if isinlong[0]: # if it's a code
                ind = isinlong[1]
            else:
                raise Exception("Question not found in the specified type!")

        else:
            raise Exception("Input data type not recognized")
    else:
        raise Exception("Invalid question type")

    return ind
```

[ ]:

```

```

Translate the questions (either a string or a list of strings) into any other format (short, long or code)

[19]:

```
def tanslateQuest(questions, in_format="Auto", out_format="Short"):
    # Translates a question from a format to another (Only Short, Long or Code)

    # questions should be either a list of strings or a string
    # The format can be only Long, Short or Code

    codes = wgm_dic["Code"]
    long = wgm_dic["Long question"]
    short = wgm_dic["Short question"]

    if type(questions) == type('abc'): # if it's a string
        questions = [questions] # make it as list

    ind_vec = list()
    out_vec = list()
```

```
    for quest in questions:
        ind = find_question_index(quest, in_format="Auto", out_format="Short")
        ind_vec.append(ind)

        if out_format == "Code":
            out = codes[ind]
            out_vec.append(out)

        elif out_format == "Short":
            out = short[ind]
            out_vec.append(out)

        elif out_format == "Long":
            out = long[ind]
            out_vec.append(out)

        else:
            raise Exception("Output format not recognized!")

    return [out_vec, ind_vec]
```

[ ]:

```

```

7.0.2 Tranlsate answers

You enter a question and it gives out the possible answers as dictionary type. Actually the real output is:

[numNval_dict, num2val, val2num]

where numNval_dict is the dictionary in both diretions (both num2val and val2num)

[20]:

```
def extractAns(question, question_in_format="Auto", question_out_format="Short",␣
 ↪ans_out_format="AShort"):
    # you can use only one question
    # Answers can be a list

    quest_index = tanslateQuest(question, in_format=question_in_format,␣
 ↪out_format="Code")[0][0]

    raw_dict = wgm_dic.loc[[quest_index], ["Ans dic"]]

    raw_dict = raw_dict.values[0][0]

    splitted = raw_dict.split(sep=', ')
#     print(splitted)

    num2val = dict()
```

```
    val2num = dict()
    numNval_dict = dict()

    for el in splitted:
        if len(el)<3:
            continue

#           print(el)
        [num, val] =el.split(sep='=')
        num = int(num)

        num2val[num] = val
        numNval_dict[num] = val

        val2num[val] = num
        numNval_dict[val] = num

    return [numNval_dict, num2val, val2num]
```

[ ]:

```

```

Translate your answes from one format to the other (you need to specify the question, of course)

[21]:

```
def translateAns(question, answers, question_in_format="Auto",␣
 ↪question_out_format="Short", ans_out_format="Auto"):
    # you can use only one question
    # Answers can be a list
    # At the moment ans_out_format can be only Auto

    trans_Ans_dict = extractAns(question, question_in_format="Auto",␣
 ↪question_out_format="Short", ans_out_format="AShort")[0]

    if not type(answers)==type(list()): # Turn the answers in a list, so we can␣
 ↪iterate
        answers = [answers]

    translated_ans = list()
    for ans in answers:
        strans_ans = trans_Ans_dict[ans]
        translated_ans.append(strans_ans)

    if len(translated_ans) == 1:
        translated_ans = translated_ans[0]

    return translated_ans
```

```
#     quest_index = tanslateQuest(question, in_format=question_in_format,␣
↪out_format="Code")[0]

#     raw_dict = wgm_dic.loc[[quest_index], ["Ans dic"]]

    return raw_dict
```

[ ]:

```

```

8 Clean the labels in the database

Make a dictionary of all the indeces -> `index_dic`

[24]:

```
# need to create a dictionary
# wgm_dic["Code"]

list_of_codes = list(wgm_dic["Code"])

index_dic = dict()

for code in list_of_codes:
    short_vers = wgm_dic.loc[[code],["Short question"]].values[0][0]

    index_dic[code] = short_vers

# index_dic
```

[ ]:

```

```

8.0.1 Make the numeric version of the database

i.e. columns names (questions) are in version short, while all the answers are numeric

-> This dataframe will be called `wgm_numeric`

[27]:

```
wgm_numeric = wgm.rename(columns=index_dic)
wgm_numeric.head()
```

[27]:

```
   Country  Nat weight     Pop weight Completion Date  Survey Year  \
0        1    0.652821  171769.597742      2018-01-08         2018
1        1    0.695706  183053.484155      2018-01-08         2018
2        1    0.523829  137829.328857      2018-01-08         2018
3        1    0.764442  201139.215039      2018-01-08         2018
4        1    3.327946  875645.512738      2018-01-08         2018

   Know Science  Understand meaning Sci  Study disease is science  \
0             3                       2                         1
```

```
1              2                        2                         1
2              2                        2                         1
3              2                        1                         1
4              2                        1                         1

   Poetry is science  Learned Sci in Prim.School  ... Age Pers Age Coho  \
0                  2                           2  ...       72        3
1                  2                           1  ...       72        3
2                 98                           1  ...       85        3
3                  2                           1  ...       54        3
4                  2                           1  ...       20        1

   Gender  Education  Area Type  Income  Region  Subjective Income  \
0       2          3          1       3       7                  2
1       1          2          2       3       7                  1
2       1          2          1       2       7                  3
3       1          3          2       5       7                  1
4       1          2          2       2       7                  1

   Income Level Employment
0             4          6
1             4          6
2             4          6
3             4          1
4             4          6

[5 rows x 60 columns]
```

[ ]:

```

```

8.0.2 Make the version with labels of the database

i.e. questions/columns as short and answers as val (not numeric)

-> wgm_labels

Note: some values are still numeric (such as the age) as it doesn't make any sense to change it. However, all the categorical questions will be changed

[28]:

```
wgm_labels = pd.DataFrame() # empty df

list_of_questions = list(wgm_dic["Short question"])
list_of_catheg_questions = list()

def translate_column(var):
    try:
        out = ans_dic[var]
    except:
```

```
        out = "Empty"
    return out

for quest in list_of_questions:

    if not wgm_dic.loc[wgm_dic["Short question"] == quest, ["Categorical Ans"]].
 ↪values[0][0]:
        # if it's not a cathegorical variable
        # Just copy it the way it is
        wgm_labels[quest] = wgm_numeric[quest]
    else:
        list_of_catheg_questions.append(quest)

        entire_col = wgm_numeric[quest]

        ans_dic = extractAns(quest)[0]

        entire_col_text = entire_col.apply(translate_column)

        wgm_labels[quest] = entire_col_text
```

[29]:
```
wgm_labels.head()
```

[29]:
```
         Country  Nat weight     Pop weight Completion Date  Survey Year  \
0  United States    0.652821  171769.597742      2018-01-08         2018
1  United States    0.695706  183053.484155      2018-01-08         2018
2  United States    0.523829  137829.328857      2018-01-08         2018
3  United States    0.764442  201139.215039      2018-01-08         2018
4  United States    3.327946  875645.512738      2018-01-08         2018

  Know Science Understand meaning Sci Study disease is science  \
0     Not much                Some of it                    Yes
1         Some                Some of it                    Yes
2         Some                Some of it                    Yes
3         Some                 All of it                    Yes
4         Some                 All of it                    Yes

  Poetry is science Learned Sci in Prim.School  ... Age Pers  Age Coho  \
0                No                         No  ...       72       50+
1                No                        Yes  ...       72       50+
2              (DK)                        Yes  ...       85       50+
3                No                        Yes  ...       54       50+
4                No                        Yes  ...       20  15 to 29

   Gender  Education                          Area Type      Income  \
0  Female   Tertiary  Lives in rural area or small town  Middle 20%
```

```
1    Male  Secondary    Lives in city or suburb of city  Middle 20%
2    Male  Secondary  Lives in rural area or small town  Second 20%
3    Male   Tertiary    Lives in city or suburb of city     Top 20%
4    Male  Secondary    Lives in city or suburb of city  Second 20%

             Region Income Level  \
0  Northern America  High income
1  Northern America  High income
2  Northern America  High income
3  Northern America  High income
4  Northern America  High income

                                 Subjective Income  \
0                       Getting by on present income
1            Living comfortably by on present income
2  Finding it difficult/very difficult to get by...
3            Living comfortably by on present income
4            Living comfortably by on present income

                         Employment
0                  Out of workforce
1                  Out of workforce
2                  Out of workforce
3  Employed full time for an employer
4                  Out of workforce

[5 rows x 60 columns]
```

[ ]:

9 Boolean version of the database (aka dummy coded)

i.e. each columns represents one combination of question and answers (e.g. “Vaccines:Trust”) the valus in the cells are then just booleans. This is useful for performing dichotomous analysis

[31]:

```
wgm_bool = pd.DataFrame()

list_of_attitudes = list()

for quest in list_of_catheg_questions: # for each question
    num2val_dic = extractAns(quest)[1]

    for key in num2val_dic: # for each anwer
        val = num2val_dic[key]
        full_str = quest+":"+val
```

```
        list_of_attitudes.append(full_str)

        col = wgm_labels[quest] == val

        wgm_bool[full_str] = col

# num2val_dic
# full_str
```

[ ]:

```
```

10 End of cleaning

Save the files to your favourite format

[33]:

```
# Excel
# filename = "wgm2018_cleaned"
# Excel_writer = pd.ExcelWriter(filename+".xlsx", engine = 'xlsxwriter')
# wgm_dic.to_excel(Excel_writer, sheet_name='Dictionary')
# wgm_numeric.to_excel(Excel_writer, sheet_name='Numeric')
# wgm_labels.to_excel(Excel_writer, sheet_name='Labels')
# wgm_bool.to_excel(Excel_writer, sheet_name='Booleans')

# Pickle
basename = "wgm2018_clean_"
wgm_dic.to_pickle(basename+"dictionary"+".pkl")
wgm_numeric.to_pickle(basename+"numeric"+".pkl")
wgm_labels.to_pickle(basename+"labels"+".pkl")
wgm_bool.to_pickle(basename+"boolean"+".pkl")

# Read
# print(pd.read_pickle(basename+"boolean"+".pkl")
```

The files are now ready to be used in the following codes

Part IV

Figure 1 and relative analysis

In this part we will generate the data and the analysis connected to figure 1: - the force directed network - the network with sets of attituteds - the conditional probability - the hierarchical clustering

[ ]:

```

```

11 Import the packages to use

[33]:

```
# import packages

import numpy as np
import pandas as pd
import matplotlib.pyplot as plt
import importlib

# import usefulFunctions as uf
# importlib.reload(uf)

from wgm2018_pack import *

import winsound
import scipy.stats as stt

from sklearn.linear_model import LinearRegression

from scipy.spatial.distance import pdist
import scipy.cluster.hierarchy as sch
import random

# Note: if you moved the package WGM_pack, make sure to uncomment the following␣
↪lines and insert the path of where you placed it
# import sys
# sys.path.insert(0, 'C:/your/path/to/the/package') # make sure to use / and not␣
↪\
```

[ ]:

```

```

12 List of attitudes

Here we set some lists that we will use later.

[2]:
```
list_science_related_attitudes2 = ['Trust neighborhood:A lot',
 'Trust neighborhood:Some',
 'Trust neighborhood:Not much',
 'Trust neighborhood:Not at all',
 'Trust government:A lot',
 'Trust government:Some',
 'Trust government:Not much',
 'Trust government:Not at all',
 'Trust Scientists:A lot',
 'Trust Scientists:Some',
 'Trust Scientists:Not much',
 'Trust Scientists:Not at all',
 'Trust Journalists:A lot',
 'Trust Journalists:Some',
 'Trust Journalists:Not much',
 'Trust Journalists:Not at all',
 'Trust Doctors:A lot',
 'Trust Doctors:Some',
 'Trust Doctors:Not much',
 'Trust Doctors:Not at all',
 'Trust NGO workers:A lot',
 'Trust NGO workers:Some',
 'Trust NGO workers:Not much',
 'Trust NGO workers:Not at all',
 'Trust science:A lot',
 'Trust science:Some',
 'Trust science:Not much',
 'Trust science:Not at all',
 'Trust Scientists 4 info:A lot',
 'Trust Scientists 4 info:Some',
 'Trust Scientists 4 info:Not much',
 'Trust Scientists 4 info:Not at all',
 'Trust scientist intentions:A lot',
 'Trust scientist intentions:Some',
 'Trust scientist intentions:Not much',
 'Trust scientist intentions:Not at all',
 'Trust scientists honesty:A lot',
 'Trust scientists honesty:Some',
 'Trust scientists honesty:Not much',
 'Trust scientists honesty:Not at all',
 'Trust scientist in Med Comp intentions:A lot',
 'Trust scientist in Med Comp intentions:Some',
 'Trust scientist in Med Comp intentions:Not much',
 'Trust scientist in Med Comp intentions:Not at all',
 'Trust scientists in Med Comp honesty:A lot',
 'Trust scientists in Med Comp honesty:Some',
 'Trust scientists in Med Comp honesty:Not much',
```

```
 'Trust scientists in Med Comp honesty:Not at all',
 'Trust gov 4 Med Advice:A lot',
 'Trust gov 4 Med Advice:Some',
 'Trust gov 4 Med Advice:Not much',
 'Trust gov 4 Med Advice:Not at all',
 'Trust Doc 4 med advice:A lot',
 'Trust Doc 4 med advice:Some',
 'Trust Doc 4 med advice:Not much',
 'Trust Doc 4 med advice:Not at all',
 'Vaccines important to children:Strongly agree',
 'Vaccines important to children:Somewhat agree',
 'Vaccines important to children:Neither agree nor disagree',
 'Vaccines important to children:Somewhat disagree',
 'Vaccines important to children:Strongly disagree',
 'Vaccines Safe:Strongly agree',
 'Vaccines Safe:Somewhat agree',
 'Vaccines Safe:Neither agree nor disagree',
 'Vaccines Safe:Somewhat disagree',
 'Vaccines Safe:Strongly disagree',
 'Vaccines Effective:Strongly agree',
 'Vaccines Effective:Somewhat agree',
 'Vaccines Effective:Neither agree nor disagree',
 'Vaccines Effective:Somewhat disagree',
 'Vaccines Effective:Strongly disagree']
```

[3]:

```
# Attitudes on vaccines
vacc_neut_att = ['Vaccines important to children:Neither agree nor disagree',␣
 ↪'Vaccines Safe:Neither agree nor disagree', 'Vaccines Effective:Neither agree␣
 ↪nor disagree']

vacc_pos_att = ['Vaccines important to children:Strongly agree',
 'Vaccines important to children:Somewhat agree','Vaccines Safe:Strongly agree',
 'Vaccines Safe:Somewhat agree','Vaccines Effective:Strongly agree',
 'Vaccines Effective:Somewhat agree']
vacc_Wpos_att = ['Vaccines important to children:Somewhat agree',
                 'Vaccines Safe:Somewhat agree', 'Vaccines Effective:Somewhat␣
 ↪agree']
vacc_Spos_att = ['Vaccines important to children:Strongly agree',
                 'Vaccines Safe:Strongly agree', 'Vaccines Effective:Strongly␣
 ↪agree']


vacc_neg_att = ['Vaccines important to children:Somewhat disagree',
 'Vaccines important to children:Strongly disagree','Vaccines Safe:Somewhat␣
 ↪disagree',
 'Vaccines Safe:Strongly disagree','Vaccines Effective:Somewhat disagree',
 'Vaccines Effective:Strongly disagree']
```

```
vacc_Wneg_att = ['Vaccines important to children:Somewhat disagree',
                 'Vaccines Safe:Somewhat disagree','Vaccines Effective:Somewhat␣
 ↪disagree']
vacc_Sneg_att = ['Vaccines important to children:Strongly disagree',
                 'Vaccines Safe:Strongly disagree','Vaccines Effective:Strongly␣
 ↪disagree']

vacc_att_full = ['Vaccines important to children:Strongly agree',
        'Vaccines Safe:Strongly agree',
           'Vaccines Effective:Strongly agree',

           'Vaccines important to children:Somewhat agree',
           'Vaccines Safe:Somewhat agree',
           'Vaccines Effective:Somewhat agree',

 'Vaccines important to children:Neither agree nor disagree',
           'Vaccines Safe:Neither agree nor disagree',
           'Vaccines Effective:Neither agree nor disagree',

 'Vaccines important to children:Somewhat disagree',
 'Vaccines Effective:Somewhat disagree',
 'Vaccines Safe:Somewhat disagree',

 'Vaccines Safe:Strongly disagree',
           'Vaccines important to children:Strongly disagree',
 'Vaccines Effective:Strongly disagree',]
```

[ ]:

```
```

[4]:

```
# Attitudes Don't know
dk_att = [
'Know Science:(DK)',
'Understand meaning Sci:(DK)',
'Study disease is science:(DK)',
'Poetry is science:(DK)',
'Learned Sci in Prim.School:(DK)',
'Learned Sci in Sec.School:(DK)',
'Learned Sci in College/Uni:(DK)',
'Searched Sci past 30d:(DK)',
'Searched Med past 30d:(DK)',
'Searched Sci:(DK)',
'Searched Med:(DK)',
'Confidence NGO:(DK)',
'Confidence Hospitals:(DK)',
'Trust neighborhood:(DK)',
'Trust government:(DK)',
'Trust Scientists:(DK)',
```

```
'Trust Journalists:(DK)',
'Trust Doctors:(DK)',
'Trust NGO workers:(DK)',
'Trust traditional Healers:(DK)',
'Trust science:(DK)',
'Trust Scientists 4 info:(DK)',
'Trust scientist intentions:(DK)',
'Trust scientists honesty:(DK)',
'Trust scientist in Med Comp intentions:(DK)',
'Trust scientists in Med Comp honesty:(DK)',
'Science benefits:(DK)',
'Science benefits you:(DK)',
'Science improve next gen:(DK)',
'Science will increase jobs:(DK)',
'Who trust most for Med Advice:(DK)',
'Trust gov 4 Med Advice:(DK)',
'Trust Doc 4 med advice:(DK)',
'Ever heard of vaccines:(DK)',
'Vaccines important to children:(DK)/(Refused)',
'Vaccines Safe:(DK)/(Refused)',
'Vaccines Effective:(DK)/(Refused)',
'Have Children:(DK)',
'Your Child Received Vax:(DK)',
'Religion:(DK)/(Refused)',
'Science disagreed w your religion:(DK)',
'(disagreement)Believe science or religion:(DK)',
]
```

[5]:

```
# Fully positive
full_trust_att = [
'Trust neighborhood:A lot',
'Trust government:A lot',
'Trust Scientists:A lot',
'Trust Journalists:A lot',
'Trust Doctors:A lot',
'Trust NGO workers:A lot',
'Trust science:A lot',
'Trust Scientists 4 info:A lot',
'Trust scientist intentions:A lot',
'Trust scientists honesty:A lot',
'Trust scientist in Med Comp intentions:A lot',
'Trust scientists in Med Comp honesty:A lot',
'Trust gov 4 Med Advice:A lot',
'Trust Doc 4 med advice:A lot',
'Confidence Hospitals:Yes',
'Science benefits you:Yes',
'Science improve next gen:Yes',
```

```
]
```

[6]:
```
# Weakly positive
medium_trus_att = [
'Trust neighborhood:Some',
'Trust government:Some',
'Trust Scientists:Some',
'Trust Journalists:Some',
'Trust Doctors:Some',
'Trust NGO workers:Some',
'Trust traditional Healers:Some',
'Trust science:Some',
'Trust Scientists 4 info:Some',
'Trust scientist intentions:Some',
'Trust scientists honesty:Some',
'Trust scientist in Med Comp intentions:Some',
'Trust scientists in Med Comp honesty:Some',
'Science benefits:Some',
'Trust gov 4 Med Advice:Some',
'Trust Doc 4 med advice:Some'
]
```

[7]:
```
# weakly negative
medium_distrust_att = [
'Trust neighborhood:Not much',
'Trust government:Not much',
'Trust Scientists:Not much',
'Trust Journalists:Not much',
'Trust Doctors:Not much',
'Trust NGO workers:Not much',
'Trust traditional Healers:Not much',
'Trust science:Not much',
'Trust Scientists 4 info:Not much',
'Trust scientist intentions:Not much',
'Trust scientists honesty:Not much',
'Trust scientist in Med Comp intentions:Not much',
'Trust scientists in Med Comp honesty:Not much',
'Trust gov 4 Med Advice:Not much',
'Trust Doc 4 med advice:Not much',
]
```

[8]:
```
# Totally negative
full_distrust_att = [
'Trust neighborhood:Not at all',
'Trust government:Not at all',
'Trust Scientists:Not at all',
'Trust Journalists:Not at all',
```

```
'Trust Doctors:Not at all',
'Trust NGO workers:Not at all',
'Trust traditional Healers:Not at all',
'Trust science:Not at all',
'Trust Scientists 4 info:Not at all',
'Trust scientist intentions:Not at all',
'Trust scientists honesty:Not at all',
'Trust scientist in Med Comp intentions:Not at all',
'Trust scientists in Med Comp honesty:Not at all',
'Trust gov 4 Med Advice:Not at all',
'Trust Doc 4 med advice:Not at all',
]
```

[9]:
```
religion_att = ['Religion:Named a specific religion']
```

[10]:
```
# Refused attitudes
refused_att = [
'Know Science:(Refused)',
'Understand meaning Sci:(Refused)',
'Study disease is science:(Refused)',
'Poetry is science:(Refused)',
'Learned Sci in Prim.School:(Refused)',
'Learned Sci in Sec.School:(Refused)',
'Learned Sci in College/Uni:(Refused)',
'Searched Sci past 30d:(Refused)',
'Searched Med past 30d:(Refused)',
'Searched Sci:(Refused)',
'Searched Med:(Refused)',
'Confidence NGO:(Refused)',
'Confidence Hospitals:(Refused)',
'Trust neighborhood:(Refused)',
'Trust government:(Refused)',
'Trust Scientists:(Refused)',
'Trust Journalists:(Refused)',
'Trust Doctors:(Refused)',
'Trust NGO workers:(Refused)',
'Trust traditional Healers:(Refused)',
'Trust science:(Refused)',
'Trust Scientists 4 info:(Refused)',
'Trust scientist intentions:(Refused)',
'Trust scientists honesty:(Refused)',
'Trust scientist in Med Comp intentions:(Refused)',
'Trust scientists in Med Comp honesty:(Refused)',
'Science benefits:(Refused)',
'Science benefits you:(Refused)',
'Science improve next gen:(Refused)',
'Science will increase jobs:(Refused)',
```

```
'Who trust most for Med Advice:(Refused)',
'Trust gov 4 Med Advice:(Refused)',
'Trust Doc 4 med advice:(Refused)',
'Ever heard of vaccines:(Refused)',
'Vaccines important to children:(DK)/(Refused)',
'Vaccines Safe:(DK)/(Refused)',
'Vaccines Effective:(DK)/(Refused)',
'Have Children:(Refused)',
'Your Child Received Vax:(Refused)',
'Religion:(DK)/(Refused)',
'Science disagreed w your religion:(Refused)',
'(disagreement)Believe science or religion:(Refused)',
]
```

[ ]:

```

```

Aggregated lists (i.e. each item is the name of a list)

[11]:

```
list_aggr_vacc = ["vacc_neut_att", "vacc_Wpos_att", "vacc_Spos_att",␣
 ↪"vacc_Wneg_att", "vacc_Sneg_att"]
list_aggr_trust = ["full_trust_att", "medium_trus_att", "medium_distrust_att",␣
 ↪"full_distrust_att"]
list_aggr_ref = ["refused_att", "dk_att"]
list_aggr_relig = ["religion_att"]
```

[ ]:

```

```

13 Functions

[12]:

```
# Calculates the correlation bootstrapping the data
def bootstrap_corr(x,y,N,f):
    l = len(x)
    c_vec = []

    for i in range(0,N):
        r = np.floor(np.random.rand(N)*l).astype(int)
        xi = x[r]
        yi = y[r]
#         xi = x
#         yi = y

        ct = f(xi,yi)

        c_vec.append(ct)

    c_vec = np.array(c_vec)
```

```
    return [c_vec, np.mean(c_vec), np.std(c_vec)]
```

[67]:
```
def spearmanr_(*args):
    # this version returns only r, which will be used for making the network
    [r,p] = stt.spearmanr(*args)

    return r
```

[13]:
```
def spearmanr_positiv(*args):
    # this version returns only r, which will be used for making the network
    [r,p] = stt.spearmanr(*args)
    if r < 0:
        r = 0

    return r
```

[15]:
```
# This is the main function for generating the network
# it takes the dataframe and calculates the edge's weight using the metrics␣
 ↪function
# exclude the same question means that it's not going to calculate correlation␣
 ↪between answers of the same question (which makes sense)
def make_graph_(list_of_nodes, metrics, exclude_same_question=True,␣
 ↪print_=False,  df=wgm_bool):
    G = nx.Graph()

    for i, node_i in enumerate(list_of_nodes):
        for j, node_j in enumerate(list_of_nodes): # for each couple of nodes

            if print_:
                n_tot = len(list_of_nodes)**2
                current = (i*len(list_of_nodes))+(j)
                print(current,"/",n_tot, " = ", np.round(current/
 ↪n_tot,decimals=2))

            if j <= i: # optimization to avoid calculating the same couple twice
                continue

            if exclude_same_question:
                if node_i.split(sep=':')[0] == node_j.split(sep=':')[0]:
                    # if they belong to the same question
                    continue

            [c1,c2] = get_col_values([node_i,node_j], df=df) # get the two␣
 ↪columns
```

```
            weight = metrics(c1,c2) # calculate the correlation
            G.add_weighted_edges_from([(node_i,node_j,weight)]) # set it in the␣
↪graph
    return G
```

[16]:
```
# Calculates partial correlation
def p_corr_(x,y,z,corr_fun):
    # correlatuon between x and y after removing z

    # Need to reshape z as column for being accepted in LinearRegression
    zz = []
    for el in z:
        zz.append([el])
    z = np.array(zz)

    # Get the residuals on x
    reg = LinearRegression().fit(z, x)
    x_predicted = reg.predict(z)
    reg = LinearRegression().fit(z, x)
    x_residuals = x-x_predicted

    # Get the residuals on y
    reg = LinearRegression().fit(z, y)
    y_predicted = reg.predict(z)
    reg = LinearRegression().fit(z, y)
    y_residuals = y-y_predicted

    p_corr = corr_fun(x_residuals, y_residuals)
    return p_corr
```

[17]:
```
# calculates partial correlation removing multiple z's
def p_corr_multiple(x,y,z_s,corr_fun):
    # z_s is a list of elements to remove
    # correlatuon between x and y after removing z

    # Need to reshape z as column for being accepted in LinearRegression

    for z in z_s:
        zz = []
        for el in z:
            zz.append([el])
        z = np.array(zz)

        # Get the residuals on x
        reg = LinearRegression().fit(z, x)
        x_predicted = reg.predict(z)
        reg = LinearRegression().fit(z, x)
```

```
        x_residuals = x-x_predicted

        # Get the residuals on y
        reg = LinearRegression().fit(z, y)
        y_predicted = reg.predict(z)
        reg = LinearRegression().fit(z, y)
        y_residuals = y-y_predicted

        x = x_residuals
        y = y_residuals

    p_corr = corr_fun(x_residuals, y_residuals)
    return p_corr
```

[18]:
```
# Just changes the shape of a 2D array
def invert_2d_array(V):
    Vp = [list() for el in V[0]]
    for el in V:
        count = 0
        for sub in el:
            Vp[count].append(sub)
            count += 1

    Vp = np.array(Vp)
    return(Vp)
```

[19]:
```
# This function acts on dataframe checking for all the people which have "at␣
↪least" one of the selected attitude
# it returns both the mask and the relative dataframe of the people who have at␣
↪least one of these attitudess
def atleast(df_, N, attitudes, PRINT=False, text=''):

    mask_t = np.array(df_[attitudes[0]]).astype(float) * 0

    for att in attitudes:
        mask_t += np.array(df_[att]).astype(float)

    mask = mask_t >= N

    l = len(df_[mask].index)

    if PRINT:
        print(text, l)

    return [mask, df_[mask], ]
```

[27]:
```python
# get the average
def get_avg_corr(G):
    nod = G.nodes

    count = 0
    avg_corr = 0

    for nn1 in nod:
        for nn2 in nod:
            count += 1
            dat = G.get_edge_data(nn1,nn2)

            if not dat == None:
                if "weight" in dat:
                    w = dat["weight"]
                    if w>=0:
                        avg_corr += w

    avg_corr = avg_corr / count

    return avg_corr
```

[31]:
```python
# shuffle the dataset while preserving the properties of the answers
# i.e. a person can only select 1 answer from the same question and no more.
def shuffle_df_for_p_exclus(df_or):
    df_new = pd.DataFrame()
    cols = df_or.columns

    l = len(df_or[df_or.columns[0]]) # len of the columns

    questions = []
    for col in cols:
        q = col.split(sep=":")[0]

        if not q in questions:
            questions.append(q)


    for q in questions:

        # select all the columns starting with this question
        col_select = []
        for col in cols:
            if q in col:
                col_select.append(col)

        one = np.ones(l)
```

```
        index_vec = list(range(0,l))
        random.shuffle(index_vec)
#           print(l)

        posit = 0

        for col in col_select: # for each column of this question

            n = np.sum(df_or[col])# number of trues

            # select the indeces
            index_select = index_vec[posit:posit+n]
            posit += n

            vec = np.zeros(l)
            vec[index_select] = 1

            df_new[col] = vec.astype(bool)

    #           print(n, np.sum(df_new[col]))

    return df_new
```

[ ]:

```

```

[ ]:

```

```

14 Making figure 1a

First, you need to generate the network

[20]:

```
# Parameters
PRINT = False # Set it to true to print the progression of the analysis
df = wgm_bool
list_of_nodes = list_science_related_attitudes2
# list_of_nodes = ['Know Science:Nothing at all', 'Trust Scientists 4 info:A␣
 ↪lot', 'Know Science:A lot']

# This is the function for generating the network
G = make_graph_(list_of_nodes, spearmanr_positiv, print_=PRINT, df=df)
# nx.draw(G, with_labels=True)
# plt.show()
```

Add the network attributes (color and size, just for visualization purposes)

[21]:
```
# Add color and size for each node (So gephi)

color_dic = {"A lot":1,"Some":2,"Not much":4,"Not at all":5,
            "Strongly agree":1,"Somewhat agree":2,"Neither agree nor disagree":3,
             "Somewhat disagree":4,"Strongly disagree":5}

attributes = dict()
for node in G.nodes:
    size = np.sum(df[node])
    color = node.split(sep=':')[1]


#     attributes[node] = {"size_":size, "color_":color}
    attributes[node] = {"size_":size, "color_":color_dic[color]}

nx.set_node_attributes(G, attributes)
```

[22]:
```
# Save
filename = 'net_data_fig_1a'
# nx.write_gexf(G,'C:/Users/DeenoZord/Documents/All_Files_Laptop/Coding/
 ↪Pyton_files/work_vaccines_limerick/Wellcome_trust/Data_analysis/
 ↪nature_full_results/gephi/'+filename+'.gexf')
# nx.write_gexf(G,'C:/where/you/want/to/save/it/'+filename+'.gexf') # <---␣
 ↪uncomment!!
```

[ ]:
```

```

Import in Gephi and run `force atlas 2` you will obtain:

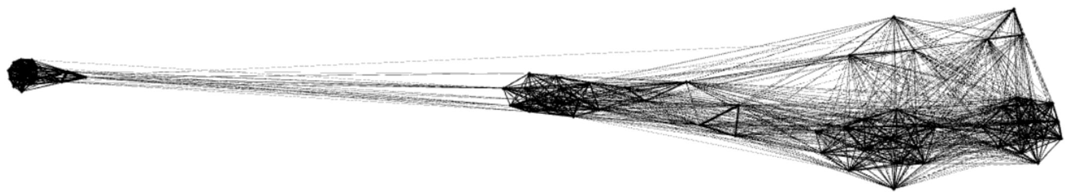

Color the nodes based on the attribute `color`

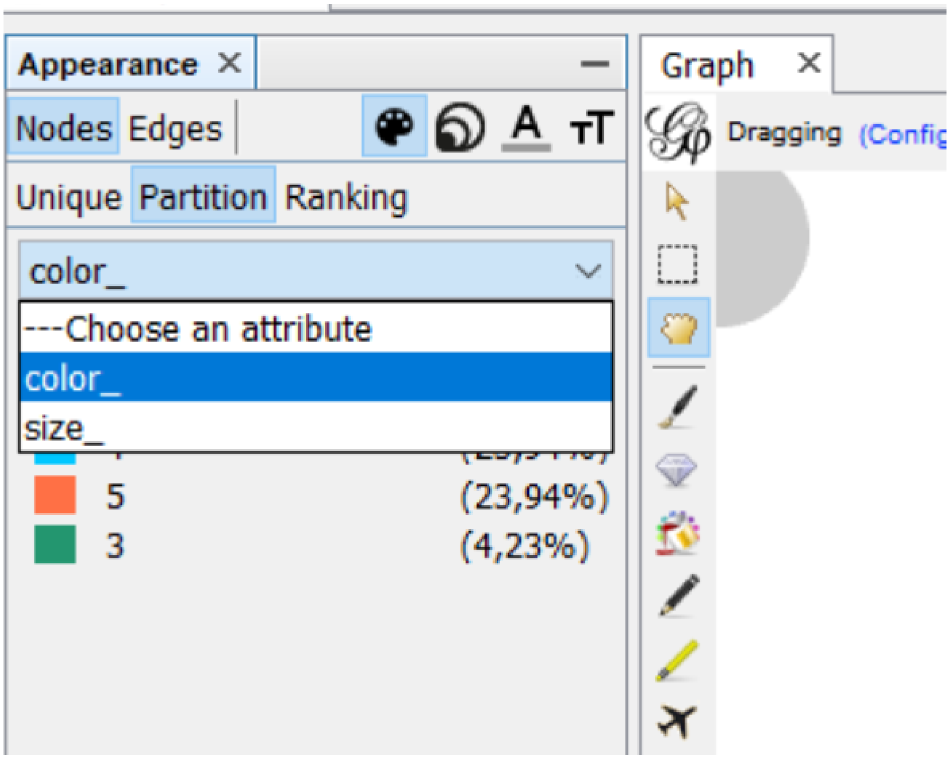

The choice of the colors is automatic by gephi, so they will not match our code of green = positive and red = negative

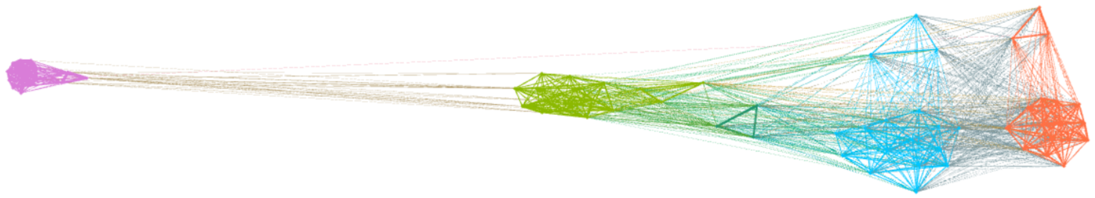

However, you can manually remap them to match the same colors. Also, you can increase the size of the nodes to make them more visible (here size 30)

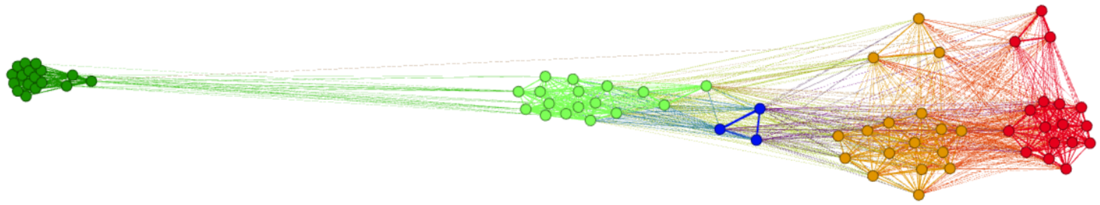

Eventually, you can also add labels and explore in details each area:

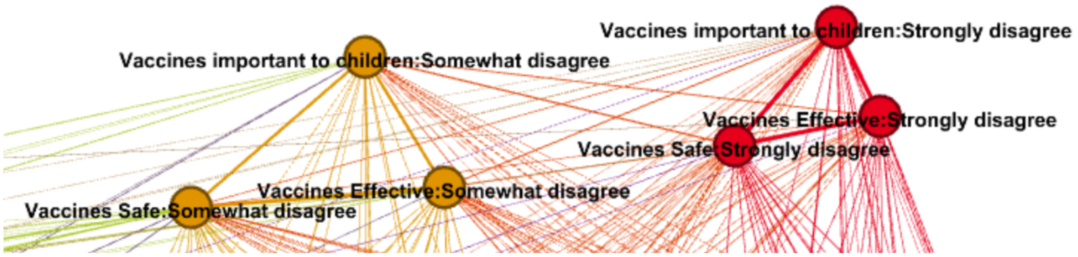

[ ]:

```
```

14.0.1 Calculate the p-value

[55]:

```
N_rep = 1000 # number of times you want to run the bootstrap to calculate the␣
↪p-value
N_sub = 1000 # if you want to use subsampling

# df_t=wgm_bool.copy() # full dataset
# df_t=wgm_bool [wgm_bool["Country:"+"Ireland"]] # subsampled on a specific␣
↪country
df_t=wgm_bool.iloc[np.random.rand(N_sub)*len(wgm_bool.iloc[:,0])]# subsampled on␣
↪N random lines

G3 = make_graph_(list_of_nodes, spearmanr_, print_=PRINT, df=df_t)

real_corr = get_avg_corr(G3)

new_corrs = []

for i in range(0, N_rep):
    print("Iteration number = ", i)

    df = shuffle_df_for_p_exclus(df_t.copy())

    Gn = make_graph_(list_of_nodes, spearmanr_, print_=False, df=df)

    corr_new = get_avg_corr(Gn)

    new_corrs.append(corr_new)
```

```
Iteration number =  0

...

Iteration number =  999
```

Analyze the results and print if it is significant or not

[57]:
```
new_corrs = np.array(new_corrs)

z_score = np.abs(real_corr-np.mean(new_corrs))/np.std(new_corrs)

print("Real corr = ", real_corr*len(G.edges))
print()

print("Max of the simulated = ", np.max(new_corrs)*len(G.edges))
print()

print("Estimated correlation")
print("mean = ", np.mean(new_corrs))
print("std = ", np.std(new_corrs))
print()

print("Significance")
print("std differences = ",z_score)

p_value = stt.norm.cdf(1-z_score)
print("p value = ",p_value)

print()
if p_value<0.01:
    print("IT IS SIGNIFICANT! :)")
else:
    print("IT IS NOT SIGNIFICANT :(")
```

```
Real corr =  108.38936524570072

Max of the simulated =  30.277789967774265

Estimated correlation
mean =  0.01189108827881595
std =  0.00024435977365493665

Significance
std differences =  138.4169404490521
p value =  0.0

IT IS SIGNIFICANT! :)
```

[ ]:
```

```

15 Make figure 1b

15.0.1 Make the second graph

Notice: this new graph includes also negative edges, so we need to recalculate `G` including the negative ones (we'll call this `G2`, while the aggregated network will be `GG`)

[58]:
```
ans_dic = {"Strongly agree":1,"Somewhat agree":2,"Neither agree nor disagree":3,
             "Somewhat disagree":4,"Strongly disagree":5}

G2 = make_graph_(list_of_nodes, spearmanr_, print_=PRINT, df=df) # Calculate G2␣
 ↪(including negative edges)

# make the network
GG = nx.Graph()
for ans in ans_dic:
    GG.add_node(ans)

for node1 in G2.nodes:
    ans1 = node1.split(sep=':')[1]
    for node2 in G2.nodes:
        ans2 = node2.split(sep=':')[1]

        if (ans1 in ans_dic) and (ans2 in ans_dic):

            if GG.get_edge_data(ans1,ans2) == None: # if the edge doesn't exist␣
 ↪yet
                GG.add_weighted_edges_from([(ans1,ans2,0)])

            if (not ans1 == ans2) and (not node1 == node2): # only if they␣
 ↪belong to two different nodes and levels
                weight0 = G2.get_edge_data(node1,node2)
                if weight0 == None:
                    weight0 = 0
                else:
                    weight0 = weight0['weight']

                weight_base = GG.get_edge_data(ans1,ans2)['weight']

                w = weight_base+weight0/9

                GG.add_weighted_edges_from([(ans1,ans2,w)])

    #     if ans in ans_dic:
    #         posit = ans_dic[ans]
    #         distrib[posit-1] += np.sum(df[node])
```

[59]:
```
# Add positivity as edge attribute

attributes = dict()
for edge in GG.edges:
    si_ = GG.get_edge_data(edge[0],edge[1])['weight']
    si_ = np.sign(si_)
    attributes[edge] = {"pos_":int(si_+2)}

nx.set_edge_attributes(GG, attributes)
```

[61]:
```
# Save
filename = 'raw_fig_1b'
# nx.write_gexf(GG,'C:/Users/DeenoZord/Documents/All_Files_Laptop/Coding/
 ↪Pyton_files/work_vaccines_limerick/Wellcome_trust/Data_analysis/
 ↪nature_full_results/gephi/'+filename+'.gexf')
# nx.write_gexf(GG,'C:/where/you/want/to/save/it/'+filename+'.gexf')
```

To make the figure, open it in gephi but do not use force atlas (or force atlas 2). Indeed, it deals very badly with negative edges. When you open it it will look like this:

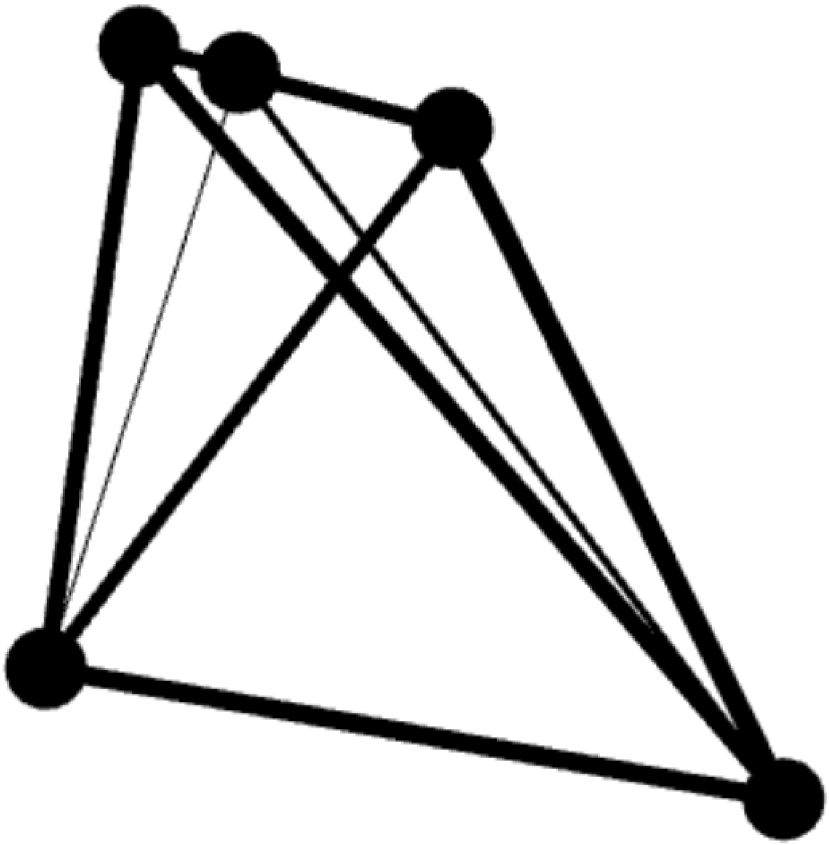

Use the edge attributes to color the edges based on their positivity. Here the value 1 would be the

negative edges and value 3 would identify

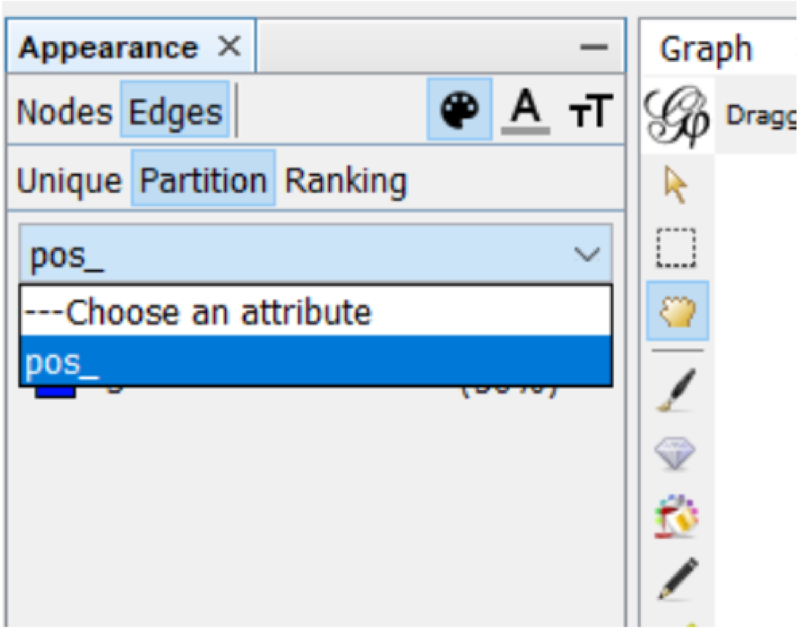

the positive ones.

Use the labels of each node to understand which one is which. Then you can manually recolor the nodes and position them in the way you prefer:

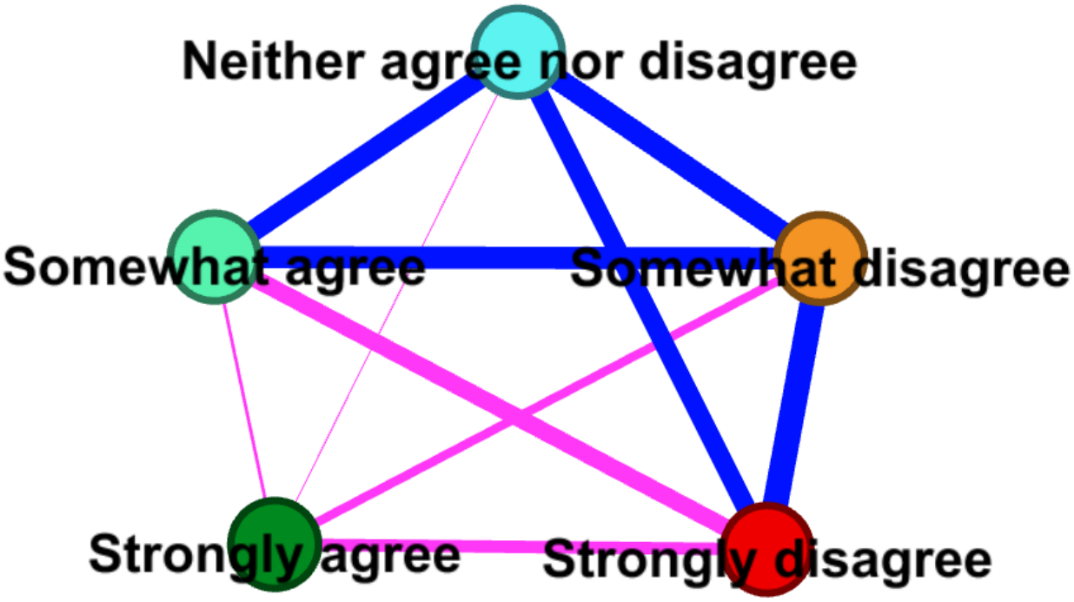

[ ]:

15.0.2 Additional visualization

If you want to remove the negative edges, you can re-run the previous analysis on G instead of G2 or directly, go to the "data laboratory" tab in gephi and remove the negative edges.

Weight	pos_
-0.27966299653053284	1
-0.248593	1
-0.157069	1
-0.103586	1
-0.025197	1
0.004106	3
0.030478	3
0.047641	3
0.0492	3
0.098509	3

If you re-run the force atlas 2 now you'll find:

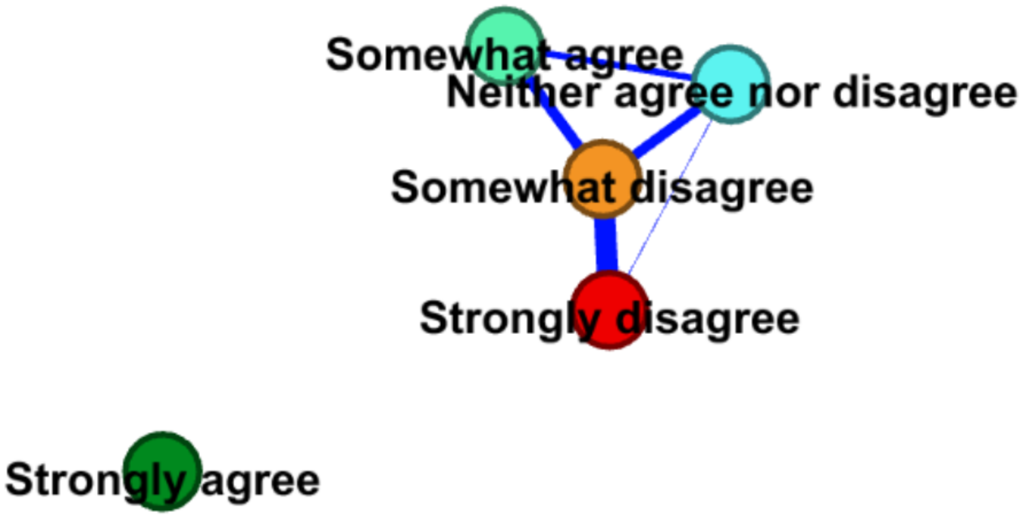

[ ]:

```
```

16 Fig 1c

Here we calculate the hierarchical clustering

[62]:

```
# Make the dendogram
df_t = wgm_bool[vacc_att_full]
arr_bool = np.array(df_t).astype(float)
```

```
arr_bool = invert_2d_array(arr_bool)

# pdist and sch are from the scipy package
y = pdist(arr_bool) #
dendogram = sch.dendrogram(sch.linkage(y, method="ward"))
```

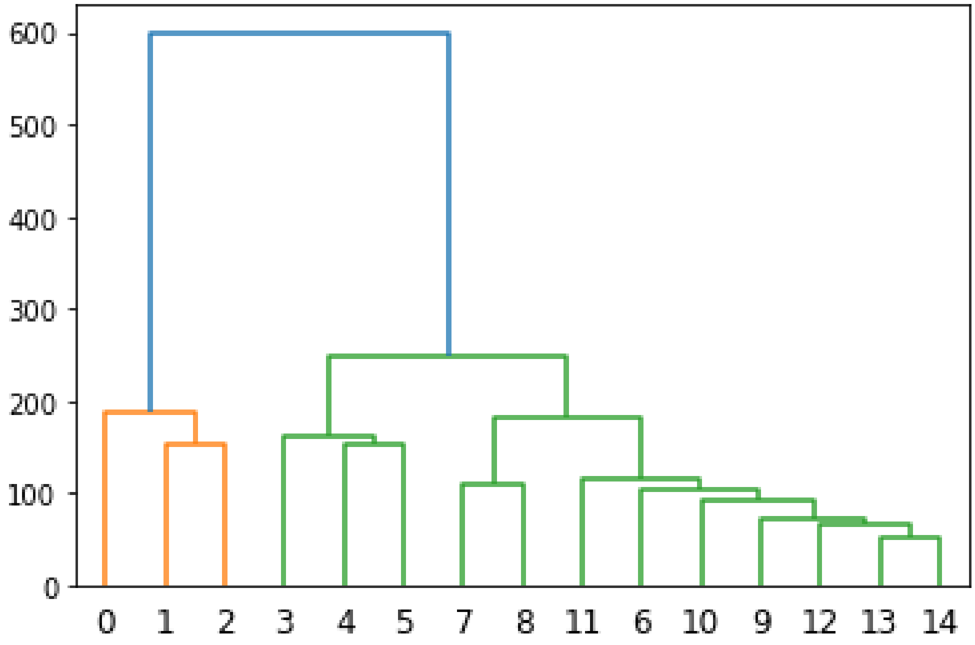

[63]:
```
# Print the legend
for i in range(0, len(vacc_att_full)):
    print(i, ' = ', vacc_att_full[i])
```

```
0  =  Vaccines important to children:Strongly agree
1  =  Vaccines Safe:Strongly agree
2  =  Vaccines Effective:Strongly agree
3  =  Vaccines important to children:Somewhat agree
4  =  Vaccines Safe:Somewhat agree
5  =  Vaccines Effective:Somewhat agree
6  =  Vaccines important to children:Neither agree nor disagree
7  =  Vaccines Safe:Neither agree nor disagree
8  =  Vaccines Effective:Neither agree nor disagree
9  =  Vaccines important to children:Somewhat disagree
10  =  Vaccines Effective:Somewhat disagree
11  =  Vaccines Safe:Somewhat disagree
12  =  Vaccines Safe:Strongly disagree
13  =  Vaccines important to children:Strongly disagree
14  =  Vaccines Effective:Strongly disagree
```

Thus we have: (dendogram + legend)

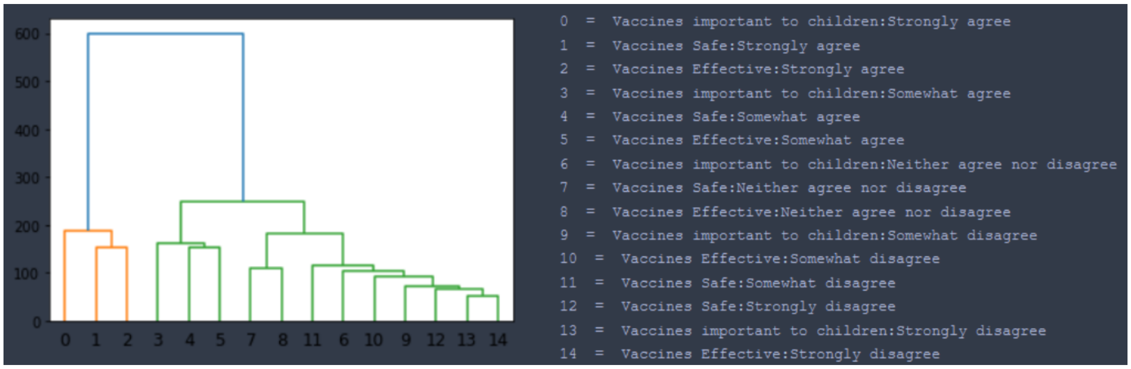

[ ]:

```

```

17 Fig 1d

Calculate the conditional probability. This is simply done by checking all the people that have at least one attitude X and then the subgroup that has also an attitude Y. Then you divide the number of people who have both an X and a Y by the number of people that have X.

This will tell you the probability that a person that has X has also Y.

[64]:

```
# The first line isolates all the people with at least 1 strongly positive␣
↪attitude
# The second checks
[M, df_t2] = atleast(wgm_bool, 1, vacc_Spos_att)
[M3, df_t3] = atleast(df_t2, 1, vacc_neut_att)
Spos_neut = np.sum(M3)/np.sum(M) # this is the conditional probability

# Repeat the same process for every level (e.g. weakly positive)
[M, df_t2] = atleast(wgm_bool, 1, vacc_Wpos_att)
[M3, df_t3] = atleast(df_t2, 1, vacc_neut_att)
Wpos_neut = np.sum(M3)/np.sum(M)

[M, df_t2] = atleast(wgm_bool, 1, vacc_neut_att)
[M3, df_t3] = atleast(df_t2, 1, vacc_neut_att)
neut_neut = np.sum(M3)/np.sum(M)

[M, df_t2] = atleast(wgm_bool, 1, vacc_Wneg_att)
[M3, df_t3] = atleast(df_t2, 1, vacc_neut_att)
Wneg_neut = np.sum(M3)/np.sum(M)

[M, df_t2] = atleast(wgm_bool, 1, vacc_Sneg_att)
[M3, df_t3] = atleast(df_t2, 1, vacc_neut_att)
Sneg_neut = np.sum(M3)/np.sum(M)

# Create two vectors with all the conditional probabilities
```

```
cond_prb1 = [Spos_neut, Wpos_neut, neut_neut, Wneg_neut, Sneg_neut]
cond_prb2 = [Spos_neut, Wpos_neut, Wneg_neut, Sneg_neut]
```

Plot the bar graph (excluding the neutrals)

[65]:
```
barlist=plt.bar(range(0,len(cond_prb2)), cond_prb2)
barlist[0].set_color('darkgreen')
barlist[1].set_color('lime')
barlist[2].set_color('orange')
barlist[3].set_color('red')
plt.grid()
```

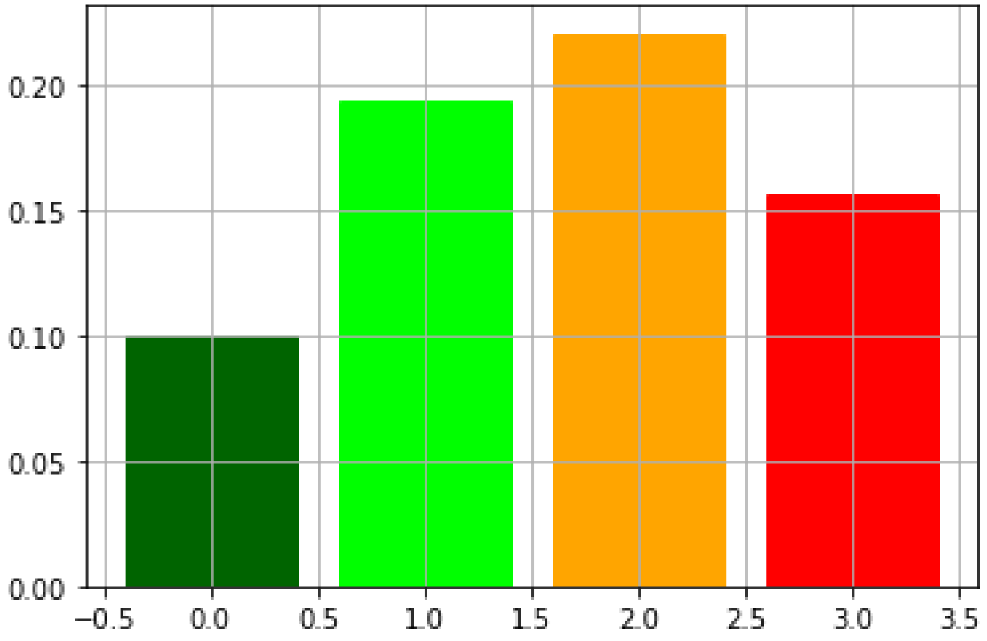

Plot the samebar graph including also the neutrals. Of course, the probability that a person with a neutral attitude has at least a neutral attitude is trivially 100%

[66]:
```
barlist=plt.bar(range(0,len(cond_prb1)), cond_prb1)
barlist[0].set_color('darkgreen')
barlist[1].set_color('lime')
barlist[3].set_color('orange')
barlist[4].set_color('red')
plt.grid()
```

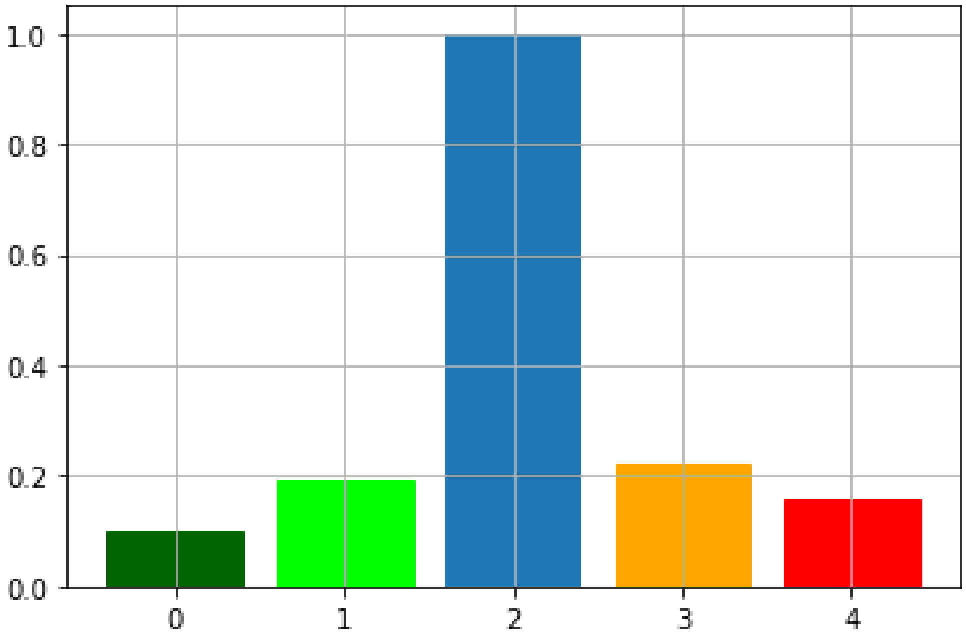

1.0
0.8
0.6
0.4
0.2
0.0
0
1
2
3
4


```
[ ]:
```

Part V

Models of social influence

This notebook is dedicated to showing how the isolation parameter affects predictions in social influence models (i.e. the more isolation, the stronger the anti-vaccine population will be in the future)

18 Import packages

[74]:
```
# import packages
# import sys
# sys.path.insert(0, 'C:/Users/DeenoZord/Documents/All_Files_Laptop/Coding/
↪Pyton_Files/Functions_and_modules')

import numpy as np
import pandas as pd
import matplotlib.pyplot as plt
import importlib

import networkx as nx
import random

import winsound

import pickle
import scipy.stats as stt
from wgm2018_pack import *

from IPython.display import clear_output
```

19 Functions & relative classes

In this first part we introduce all the functions to be used later. Many of them are used to provide a general framework for all the simulations. In such a way that the differences between models would be because of the formal model differences and not because each model has been written in a different way. In these models we have the following types of rules and properties: - network = the structure of the network for interaction (here a fully connected one) - Selection rule = how agents select each other for the interaction - update rule = how agents change opinion after interaction - tick = starting a new process of interaction

19.0.1 Special functions

For dictionaries

[75]:
```
# Calculates the mean from the values of a dictionary
def dic_mean(dic):
    vals_ = [dic[key] for key in dic]
    vals_ = np.array(vals_)
    return np.mean(vals_)
```

[76]:
```
def dic2array(dic):
    x = []
    y = []
    for key in dic:
        x.append(key)
        y.append(dic[key])

    return [x,y]
```

[77]:
```
def array2dic(x,y):
    dic = dict()

    for i in range(0,len(x)):
        xi = x[i]
        yi = y[i]

        dic[xi] = yi

    return dic
```

Save and read

[78]:
```
def save_obj(obj, name ):
    with open(name + '.pkl', 'wb') as f:
        pickle.dump(obj, f, pickle.HIGHEST_PROTOCOL)

def load_obj(name ):
    with open(name + '.pkl', 'rb') as f:
        return pickle.load(f)
```

Other

[79]:
```
def get_index_below_thr(dic, thr):

    dic2 = dict()

    for key in dic:
        el = dic[key]

        if el <= thr:
            dic2[key] = el
    return dic2
```

[80]:
```
def beep():
    freq = 500
    dur = 200
    winsound.Beep(freq, dur)

    return
```

19.0.2 Functions for labelling

[81]:
```
# Initialize the names of the agents as a set of numbers
def ordered_numbers_(self=None, N=10):
    agents_list = [str(i) for i in range(0,N)]
    if not (self==None):
        self.labels = agents_list
    else:
        return agents_list
```

Class containing all

[82]:
```
class generate_labels:
    ordered_numbers = ordered_numbers_
#     bb = number_naming
```

19.0.3 Functions for the Network

[83]:
```
def mk_lattice_(self=None, dim=10,labels=None,periodic=False):
    # if dim is a single number it will make a square lattice dim*dim
    if self!= None:
        labels = self.labels

    if type(dim) == type(1):
        dim = [dim, dim]

    G = nx.grid_graph(dim=dim,periodic=periodic)

    # Rename the nodes with the right names
    nodes = list(G.nodes)
    d = {el:labels[i] for i, el in enumerate(nodes)}
    G = nx.relabel_nodes(G,d)

    if self!= None:
        self.network = G
    else:
        return G
```

class

[84]:
```
class mk_network:
    mk_lattice = mk_lattice_
```

19.0.4 Functions for Initializing opinions

[85]:
```
# Initialize the agents with random opinions from min and max [uniform␣
 ↪distribution]
def rand_opinions_(self=None, labels=None, min_=0, max_=1):
    if self!=None:
        labels = self.labels
#     else:
#         print("self not passed")

    N = len(labels)
    op_vec = (np.random.rand(N)*(max_-min_))+min_
    dic_op = {labels[i]:op_vec[i] for i in range(0,N)}

    if self!=None:
        self.opinions = dic_op
    else:
        return dic_op
```

[86]:
```
# Initialize from a specific distribution
# x_distrib represents the value, while
# y_distrib represents their distribution
def op_from_distrib_(self=None, x_distrib=[], y_distrib=[], labels=None):
    if self!=None:
        labels = self.labels

    dic_op = {}

    N = len(labels)

    # renormalize y
    y_distrib = np.array(y_distrib)
    y_distrib = y_distrib/np.sum(y_distrib)

    index_distrib = list(range(0,len(x_distrib)))

    for i in range(0,N):
        found = False

        while not found:

            ii = random.choice(index_distrib)
```

```
            xi = x_distrib[ii]
            yi = y_distrib[ii]

            r = np.random.rand()

            if yi>r:
                found = True
                dic_op[labels[i]] = xi

    if self!=None:
        self.opinions = dic_op
    else:
        return dic_op
```

[87]:
```
# Initialize from a specific distribution
# x_distrib represents the value, while
# y_distrib represents their distribution
def axel_from_distrib_(self=None, x_distrib=[], y_distrib=[], labels=None):
    if self!=None:
        labels = self.labels

    dic_op = {}

    N = len(labels)

    # renormalize y
    y_distrib = np.array(y_distrib)
    y_distrib = y_distrib/np.min(y_distrib[y_distrib>0])
    y_distrib = np.round(y_distrib)

    x_distrib2 = []
    for i, xi in enumerate(x_distrib):
        yi = int(y_distrib[i])
        x_distrib2 = x_distrib2 + ([xi]*yi)

    for i in range(0,N):
        dic_op[labels[i]] = random.choice(x_distrib2)


    if self!=None:
        self.opinions = dic_op
    else:
        return dic_op
```

[88]:
```
def rand_op_axel_(self=None, labels=None, f=3, q=5):
    if self!=None:
        labels = self.labels
```

```
    ff = f-0.0001
    dic_op = dict()

    for ag in labels:
        op = np.floor(np.random.rand(q)*ff)
        dic_op[ag] = op

    if self!=None:
        self.opinions = dic_op
    else:
        return dic_op
```

Class

[89]:
```
class initialize_opinions:
    rand_opinions = rand_opinions_
    rand_op_axel = rand_op_axel_
    op_from_distrib = op_from_distrib_
    axel_from_distrib = axel_from_distrib_
```

19.0.5 Selection rules

i.e. how agents select each other

[90]:
```
def select_random_(self=None, lab=None):
    if self!= None:
        lab = self.labels

    lab_c = lab.copy()
    ag1 = random.choice(lab_c)
    lab_c.remove(ag1) # remove it so it won't be choosen for ag2

    ag2 = random.choice(lab_c)

    if self!= None:
        self.selected_agents =[ag1,ag2]
    else:
        return [ag1,ag2]
```

[91]:
```
def select_deff_random_(self=None, lab=None, opinions= None, eps=None):
    # Note: if it's not able to find an element in the range it'll give back a␣
 ↪none

    if self!= None:
        lab = self.labels
        eps = self.parameters["eps"]
```

```
        opinions = self.opinions

    lab_c = lab.copy()
#     print(labels)
    ag1 = random.choice(lab_c)
    op1 = opinions[ag1]

    lab_c.remove(ag1) # remove it so it won't be choosen for ag2

    over = False

    while not over:
        if len(lab_c) > 1:
            ag2 = random.choice(lab_c)
            op2 = opinions[ag2]
            if np.abs(op2-op1) <= eps:
                over = True
            else:
                try:
                    lab_c.remove(ag2)
                except:
                    op2 = None
                    over = True
        else:
            op2 = None
            over = True

    if self!= None:
        self.selected_agents =[ag1,ag2]
    else:
        return [ag1,ag2]
```

```
[92]: # The first agent is selected randomly and then it selects all the other within␣
       ↪epsilon
def select_HK_random_(self=None, lab=None, opinions=None, eps=None):
    # Note: if it's not able to find an element in the range it'll give back a␣
       ↪none

    if self!= None:
        lab = self.labels
        eps = self.parameters["eps"]
        opinions = self.opinions

    lab_c = lab.copy()
#     print(labels)
    ag1 = random.choice(lab_c)
    op1 = opinions[ag1]
```

```
    ag_others = [ag for ag in opinions if np.abs(opinions[ag]-op1)<=eps]
    ag_others.append(ag1)

    if self!= None:
        self.selected_agents = ag_others
    else:
        return ag_others
```

[93]:
```
def select_neigh_(self=None, labels=None, net=None):
    if self!= None:
        lab = self.labels
        net = self.network

    # randomly choose the first agent
    ag1 = random.choice(labels)

    neigh = net.neighbors(ag1)

    # choose the second from the neighbours
    ag2 = random.choice(neigh)

    if self!= None:
        self.selected_agents =[ag1,ag2]
    else:
        return [ag1,ag2]
```

Class

[94]:
```
class make_selection_rule:
    select_random = select_random_
    select_neigh = select_neigh_
    select_deff_random = select_deff_random_
    select_HK_random = select_HK_random_
```

19.0.6 Update rules

i.e. how agents change opinion after interaction

[95]:
```
def average_(self=None, selected_agents=None, opinions=None):
    # suppose selected are 2 for now
    if self!=None:
        selected_agents = self.selected_agents # this is a list
        opinions = self.opinions # this is a dic


    selected_op = [opinions[key] for key in selected_agents]
```

```
    avg = np.mean(np.array(selected_op))

    # replace the values
    if self!=None:
        for key in selected_agents:
            self.opinions[key] = avg
    else:
        for key in selected_agents:
            opinions[key] = avg
        return opinions
```

[96]:
```
def average_deff_(self=None, selected_agents=None, opinions=None, mu=None):
    # suppose selected are 2 for now
    if self!=None:
        selected_agents = self.selected_agents # this is a list
        opinions = self.opinions # this is a dic
        mu = self.parameters["mu"]


    selected_op = [opinions[key] for key in selected_agents]
    avg = np.mean(np.array(selected_op))

    selected_op2 = {key:(opinions[key]*(1-2*mu)+avg*2*mu) for key in␣
 ↪selected_agents}

    # replace the values
    if self!=None:
        for key in selected_agents:
            self.opinions[key] = selected_op2[key]
    else:
        for key in selected_agents:
            opinions[key] = selected_op2[key]
        return opinions
```

[97]:
```
def geo_mean_(self=None, selected_agents=None, opinions=None):
    # suppose selected are 2 for now
    if self!=None:
        selected_agents = self.selected_agents # this is a list
        opinions = self.opinions # this is a dic


    selected_op = [opinions[key] for key in selected_agents]
    avg = stt.mstats.gmean(np.array(selected_op))

    # replace the values
```

```python
    if self!=None:
        for key in selected_agents:
            self.opinions[key] = avg
    else:
        for key in selected_agents:
            opinions[key] = avg
        return opinions
```

[98]:
```python
def h_mean_(self=None, selected_agents=None, opinions=None):
    # suppose selected are 2 for now
    if self!=None:
        selected_agents = self.selected_agents # this is a list
        opinions = self.opinions # this is a dic


    selected_op = [opinions[key] for key in selected_agents]
    avg = stt.mstats.hmean(np.array(selected_op))

    # replace the values
    if self!=None:
        for key in selected_agents:
            self.opinions[key] = avg
    else:
        for key in selected_agents:
            opinions[key] = avg
        return opinions
```

[99]:
```python
def copy_trait_(self=None, selected_agents=None, opinions=None):
    # suppose selected are 2 for now
    if self!=None:
        selected_agents = self.selected_agents # this is a list
        opinions = self.opinions # this is a dic

    op1 = np.array(opinions[selected_agents[0]])
    op2 = np.array(opinions[selected_agents[1]])

    unshered_op_mask = op1!=op2
    indeces = np.array(range(0,len(op1)))
    indeces = indeces[unshered_op_mask]

    if len(indeces) > 0:
        i = random.choice(indeces)
        op2[i] = op1[i]

        if self!=None:
            self.opinions[selected_agents[0]] = op1
            self.opinions[selected_agents[1]] = op2
```

```
[100]: def copy_trait_distance_(self=None, selected_agents=None, opinions=None):
    # suppose selected are 2 for now

    if self!=None:
        selected_agents = self.selected_agents # this is a list
        opinions = self.opinions # this is a dic

    op1 = np.array(opinions[selected_agents[0]])
    op2 = np.array(opinions[selected_agents[1]])

    rel_distance = np.abs(np.sum(op1)-np.sum(op2))/self.
 ↪parameters["max_distance"]
    proximity = (np.sum(op1==op2))/len(op1) * (1-rel_distance)**0
#     print("prox", proximity )

    r = np.random.rand()

    if r < proximity:
        unshered_op_mask = op1!=op2
        indeces = np.array(range(0,len(op1)))
        indeces = indeces[unshered_op_mask]

        if len(indeces) > 0:
            i = random.choice(indeces)
            op1[i] = op2[i]

            if self!=None:
                self.opinions[selected_agents[0]] = op1
                self.opinions[selected_agents[1]] = op2
```

Class

```
[101]: class make_interaction:
    average = average_
    copy_trait = copy_trait_
    average_deff = average_deff_
    geo_mean = geo_mean_
    h_mean = h_mean_
```

19.0.7 Observables

[102]:
```
def op_dic_(self=None, opinions=None):

    if self!=None:
        opinions = self.opinions

    if self!=None:
        self.observables['opinions'] = opinions
    else:
        return opinions
```

[103]:
```
def op_arr_(self=None, opinions=None):

    if self!=None:
        opinions = self.opinions

    if self!=None:
        self.observables['opinions'] = dic2array(opinions)[1]
    else:
        return opinions
```

[104]:
```
def average_op_(self=None, opinions=None):

    if self!=None:
        opinions = self.opinions

    avg = np.mean(np.array(list(opinions.values())))

    if self!=None:
        self.observables['avg'] = avg
    else:
        return avg
```

[105]:
```
def sum_op_(self=None, opinions=None):
    if self!=None:
        opinions = self.opinions

    sum_ = np.sum(np.array(list(opinions.values())))

    if self!=None:
        self.observables['sum'] = sum_
    else:
        return sum_
```

[106]:
```
def frequency_op_(self=None, opinions=None):
    if self!=None:
```

```
        op_dic = self.opinions

    freq = dict()
    for key in op_dic:
        el = op_dic[key]

        if el in freq:
            freq[el] += 1
        else:
            freq[el] = 1

    if self!=None:
        self.observables['frequency_op'] = freq
    else:
        return freq
```

class

[107]:
```
class make_observables:
    average_op = average_op_
    sum_op = sum_op_
    op_arr = op_arr_
    op_dic = op_dic_
    frequency_op = frequency_op_
```

20 Functions for running and ticking

20.0.1 Ticks

[108]:
```
def tick_classic_(self):
#     self.selec_rule(self)
#     self.up_rule(self)
    self.selec_rule()
    self.up_rule()
```

class

[109]:
```
class ticks:
    tick_classic = tick_classic_
```

20.0.2 Run

[110]:
```
def run_N_times_(self=None,N=None):
    if self!=None:
        N = self.times_2_run
```

```
    for i in range(0,N):
        self.tick()
```

Class

[111]:
```
class run:
    run_N_times = run_N_times_
```

21 Main class

[112]:
```
class model:
    observables = dict()
    has_converged = False
    times_2_run = 0
    pass
```

[ ]:
```
```

22 Functions for dealing with the data

The following functions are used to deal with the fact that the vaccine data are based on 3 questions, while several models require only 1 opinion value. However, some models, such as the Axelrod one, work well with the three different questions. So we need to create a framework which can work with both of them

[113]:
```
def make_aggregate_prb(coeff_val=[0,1,2,3,5]): # this works directly with the␣
 ↪Vaccine data

    dic_prb2 = dict()

    for raw_vec in dic_probability:
        prb = dic_probability[raw_vec]

        new_vec = transform_raw_vec(vec=raw_vec, coeff_val=coeff_val)

        sum_ = np.sum(new_vec)

        if sum_ not in dic_prb2:
            dic_prb2[sum_] = prb
        else:
            dic_prb2[sum_] += prb

    return dic_prb2
```

[114]:
```python
def transform_raw_vec(vec='[0, 0, 4]', coeff_val=[0,1,2,3,5]):
#     print(vec)
    v_t = [int(vec[1]),int(vec[4]),int(vec[7])]

    v_2 = []
    for el in v_t:
        v_2.append(coeff_val[el])

    v_2 = np.array(v_2)

    return v_2

transform_raw_vec()
```

[114]:
```
array([0, 0, 5])
```

[115]:
```python
def standardize_score(score, min_,max_, mean_):
    try:
        len(score)
        is_array = True
    except:
        is_array = False

    if is_array:
        score2 = np.array(score)
        score2 = score2-mean_
        score2[score2>0] = score2[score2>0] / (max_-mean_)
        score2[score2<0] = score2[score2<0] / (mean_-min_)
    else:
        score2 = score
        score2 = score2-mean_
        if score2 > 0:
            score2 = score2 / (max_-mean_)
        else:
            score2 = score2 / (mean_-min_)

    return score2
```

[116]:
```python
# replaces 0 with 4 etc in such a way that 0 indicates anti-vax and 4 pro-vax
def invert_dic(dic_):

    dt_ = dict()

    for key in dic_:
        k = key
```

```
        k = k.replace('0','a')
        k = k.replace('1','b')
        k = k.replace('2','c')
        k = k.replace('3','d')
        k = k.replace('4','e')

        k = k.replace('a','4')
        k = k.replace('b','3')
        k = k.replace('c','2')
        k = k.replace('d','1')
        k = k.replace('e','0')

        dt_[k] = dic_[key]

    return dt_
```

[ ]:

```

```

23 Lists

[117]:

```
att_list = [['Vaccines important to children:Strongly agree',
 'Vaccines important to children:Somewhat agree',
 'Vaccines important to children:Neither agree nor disagree',
 'Vaccines important to children:Somewhat disagree',
 'Vaccines important to children:Strongly disagree'],
 ['Vaccines Safe:Strongly agree',
 'Vaccines Safe:Somewhat agree',
 'Vaccines Safe:Neither agree nor disagree',
 'Vaccines Safe:Somewhat disagree',
 'Vaccines Safe:Strongly disagree'],
 ['Vaccines Effective:Strongly agree',
 'Vaccines Effective:Somewhat agree',
 'Vaccines Effective:Neither agree nor disagree',
 'Vaccines Effective:Somewhat disagree',
 'Vaccines Effective:Strongly disagree']]

countries = countries_list
```

[118]:

```
list_aggr_vacc2 = ['vacc_neut_att',
 'vacc_Wpos_att',
 'vacc_Wneg_att',
 'vacc_Sneg_att']

list_aggr_vacc_Spos = ['vacc_Spos_att']
```

```
list_aggr_vacc = ["vacc_neut_att", "vacc_Wpos_att", "vacc_Spos_att",␣
 ↪"vacc_Wneg_att", "vacc_Sneg_att"]
list_aggr_trust = ["full_trust_att", "medium_trus_att", "medium_distrust_att",␣
 ↪"full_distrust_att"]
list_aggr_ref = ["refused_att", "dk_att"]
list_aggr_relig = ["religion_att"]
```

[119]:
```
vacc_neut_att = ['Vaccines important to children:Neither agree nor disagree',␣
 ↪'Vaccines Safe:Neither agree nor disagree', 'Vaccines Effective:Neither agree␣
 ↪nor disagree']

vacc_pos_att = ['Vaccines important to children:Strongly agree',
 'Vaccines important to children:Somewhat agree','Vaccines Safe:Strongly agree',
 'Vaccines Safe:Somewhat agree','Vaccines Effective:Strongly agree',
 'Vaccines Effective:Somewhat agree']
vacc_Wpos_att = ['Vaccines important to children:Somewhat agree',
                 'Vaccines Safe:Somewhat agree', 'Vaccines Effective:Somewhat␣
 ↪agree']
vacc_Spos_att = ['Vaccines important to children:Strongly agree',
                 'Vaccines Safe:Strongly agree', 'Vaccines Effective:Strongly␣
 ↪agree']


vacc_neg_att = ['Vaccines important to children:Somewhat disagree',
 'Vaccines important to children:Strongly disagree','Vaccines Safe:Somewhat␣
 ↪disagree',
 'Vaccines Safe:Strongly disagree','Vaccines Effective:Somewhat disagree',
 'Vaccines Effective:Strongly disagree']
vacc_Wneg_att = ['Vaccines important to children:Somewhat disagree',
                 'Vaccines Safe:Somewhat disagree','Vaccines Effective:Somewhat␣
 ↪disagree']
vacc_Sneg_att = ['Vaccines important to children:Strongly disagree',
                 'Vaccines Safe:Strongly disagree','Vaccines Effective:Strongly␣
 ↪disagree']
```

[ ]:
```
```

24 Initialize the data

Calculate the distribution of opinions for each country

[120]:
```
distributions_dic = dict()

for country in countries:
    country_mask = wgm_labels["Country"] == country
    df_country = wgm_bool.copy()
```

```
    df_country = df_country[country_mask]

    dict_t = dict()

    max_ = 0

    for j1 in range(0,4+1):
        for j2 in range(0,4+1):
            for j3 in range(0,4+1):
                vec = [j1, j2, j3]

                selected_atts = [att_list[0][j1], att_list[1][j2],␣
 ↪att_list[2][j3]]

                m1 = df_country[selected_atts[0]]
                m2 = df_country[selected_atts[1]]
                m3 = df_country[selected_atts[2]]

                mm = m1 & m2 & m3
#                 print(mm)

                sum_ = np.sum(mm)
                dict_t[str(vec)] = sum_

                if max_ < sum_:
                    max_ = sum_

    for key in dict_t:
        dict_t[key] = dict_t[key]/max_

    distributions_dic[country] = dict_t
```

[121]:
```
def spearmanr_(*args):
    # this version returns only r, which will be used for making the network
    [r,p] = stt.spearmanr(*args)

    return r
```

[122]:
```
def check_avg_corr(list_of_aggregated_nodes, list_of_aggregated_nodes_n,␣
 ↪metrics, exclude_same_question=True, print_=False,  df=wgm_bool):
    # The first aggregated node is the one that you'll use to calculate the␣
 ↪correlation

#     print(list_of_aggregated_nodes_n)
    a_node_i_name = list_of_aggregated_nodes_n[0]
    a_node_i = list_of_aggregated_nodes[0]
```

```
    count = 0
    for j, a_node_j_name in enumerate(list_of_aggregated_nodes_n):
        if j == 0:
            pass
        else:
            a_node_j = list_of_aggregated_nodes[j]

    #         count = 0
            avg_weight = 0
    #             print(a_node_i)
            for ii, node_i in enumerate(a_node_i):
                for jj, node_j in enumerate(a_node_j):

    #                         if print_:
    #                             n_tot = len(list_of_nodes)**2
    #                             current = (i*len(list_of_nodes))+(j)
    #                             print(current,"/",n_tot, " = ", np.
↪round(current/n_tot,decimals=2))

                    if exclude_same_question:
                        if node_i.split(sep=':')[0] == node_j.split(sep=':')[0]:
                            # if they belong to the same question
                            continue

                    [c1,c2] = get_col_values([node_i,node_j],df=df)
                    weight = metrics(c1,c2)

                    if not np.isnan(weight):
                        avg_weight = (weight + avg_weight*count)/(count+1)
                        count += 1

    return avg_weight
```

25 Calculate the isolation parameter for each country

[123]:
```
df_tot = wgm_bool

df = df_tot

country_corr_dict = dict()

exclude_same_question = True # True or false

metrics = spearmanr_
list_of_aggregated_nodes_n = list_aggr_vacc_Spos+list_aggr_vacc2 # Best
```

```
list_of_aggregated_nodes = []
for name in list_of_aggregated_nodes_n:
    exec("t = "+name)
    list_of_aggregated_nodes.append(t)

########################################################################
no_vax_score_vec = []
corr_score_vec = []
n_notSpos = []
n_Spos = []

# For each country in the df
country_list_df = [col for col in df.columns if "Country:" in col] # select the␣
↪country columns
country_list_df = [col for col in country_list_df if np.sum(df[col]) > 0]  #␣
↪select the ones that have no 0 elements

country_count = 0
tot_country = len(country_list_df)
for count_col in country_list_df:
    country_count += 1
#     print(country_count, " out of ", tot_country)

    df_count = df [ df[count_col] == 1]
    avg_corr = check_avg_corr(list_of_aggregated_nodes,␣
↪list_of_aggregated_nodes_n, metrics,␣
↪exclude_same_question=exclude_same_question, print_=False,  df=df_count)

    if (not np.isnan(avg_corr)):
        country_corr_dict[count_col.split(':')[1]] = avg_corr

    clear_output()
```

[ ]:

```

```

26 Run the models

Now we run each model on the WGM data and see how they affect the skeptic people

26.0.1 Deffuant

Firstly define the deffuant model by specifying all the rules

[51]:

```
class deffuant_alt1(model):
    # initialization
```

```
    make_lab = generate_labels.ordered_numbers
    init_op = initialize_opinions.op_from_distrib

    # rules
    selec_rule = make_selection_rule.select_deff_random
    up_rule = make_interaction.average_deff

    # observables
    mk_ob_op = make_observables.op_dic
    mk_ob_avg = make_observables.average_op
    mk_op_frq = make_observables.frequency_op

    # Run
    tick = ticks.tick_classic
    run = run.run_N_times
```

[52]:
```
deff = deffuant_alt1()
```

Determine the parameters (eps is the threshold)

[53]:
```
    # parameters
N_agents = 1000
Run_times = 20 # each agent will be run, on average this number of time!
deff.parameters = {"mu":0.5, "eps":3, "N_agents":N_agents}
deff.times_2_run = N_agents*Run_times
```

Run the model

[54]:
```
# Lists for storing the simulated data
initial_score = []
final_score = []
correlation_score = []

repetitions = 1000 # This determines the number of simulated countries (i.e. the␣
↪number of points in the plot)

dic_pred_deff = {}

for rr in range(0,repetitions):

    # Select one random country
    country = random.choice(list(distributions_dic.keys()))
    country_corr = country_corr_dict[country]

    # Make the dictionary with the prb of answers
    dic_probability = distributions_dic[country] # this is the dictionary that␣
↪will be used for cheching the probabilities
```

```python
    dic_probability = invert_dic(dic_probability) # invert the dic probability␣
↪(e.g. 4 to 0)

    deff.make_lab(N=N_agents) # initialize the model

    value_of_answers = [0,1,2,3,4] # specify the type of answers

    dd = make_aggregate_prb(coeff_val=np.array(value_of_answers)) # turn the␣
↪answers into a single score

    x = []
    y = []
    for key in dd:
        x.append(key)
        y.append(dd[key])

    deff.init_op(x_distrib=x, y_distrib=y)

    # Find the skeptics and their initial score
    min_ = np.min(x)
    mean_= value_of_answers[2]*3
    max_ = np.max(x)

    threshold = mean_ # below this value you are considered skeptic

    initial_below_thr = get_index_below_thr(deff.opinions, thr=threshold)
    avg1 = dic_mean(initial_below_thr)
    labels_initial_below = [key for key in initial_below_thr]

    score0 = standardize_score(avg1, min_, max_, mean_)

    # Run
    deff.run()

    # Get the data from the end of the simulation
    op_dic = deff.opinions
    # Get the agents which were initially skeptics
    new_below_thr = {key:op_dic[key] for key in labels_initial_below}

    avg1 = dic_mean(new_below_thr)
    score_fin = standardize_score(avg1, min_, max_, mean_)

    if not np.isnan(score_fin):
        initial_score.append(score0)
        final_score.append(score_fin)
        correlation_score.append(country_corr)
        dic_pred_deff[country] = score_fin
```

```
beep()
```

[55]:
```
x = np.array(correlation_score)
y = np.array(final_score)

plt.scatter(-x,y)
plt.xlabel("Isolation")
plt.ylabel("Final posit of the skeptics")
plt.grid()

print(stt.spearmanr(-x,y))
```

```
SpearmanrResult(correlation=-0.4242329619819221, pvalue=5.926421219012715e-45)
```

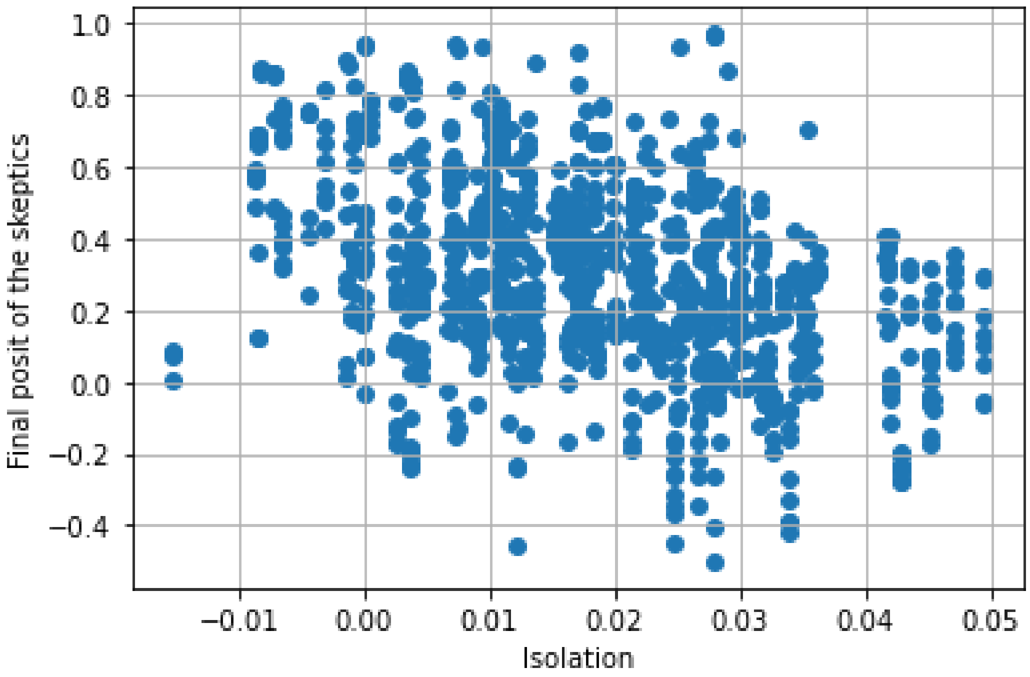

[ ]:
```

```

26.0.2 HK (standard mean)

i.e. Hegselmann Krause model with standard mean function

Define the the model

[56]:
```
class HK(model):
    # initialization
```

```
    make_lab = generate_labels.ordered_numbers
    init_op = initialize_opinions.op_from_distrib

    # rules
    selec_rule = make_selection_rule.select_HK_random
    up_rule = make_interaction.average

    # observables
    mk_ob_op = make_observables.op_dic
    mk_ob_avg = make_observables.average_op
    mk_op_frq = make_observables.frequency_op

    # Run
    tick = ticks.tick_classic
    run = run.run_N_times
```

[57]:
```
HK1 = HK()
```

Define the parameters

[58]:
```
# parameters
N_agents = 1000
HK1.parameters = {"eps":3, "N_agents":N_agents}
HK1.times_2_run = 100
```

Run the simulation

[59]:
```
initial_score = []
final_score = []
correlation_score = []

repetitions = 1000

dic_HK1_deff = {}

for rr in range(0,repetitions):

    # Select one random country
    country = random.choice(list(distributions_dic.keys()))
    country_corr = country_corr_dict[country]

    # Make the dictionary with the prb of answers
    dic_probability = distributions_dic[country] # this is the dictionary that␣
↪will be used for cheching the probabilities
    dic_probability = invert_dic(dic_probability)


    HK1.make_lab(N=N_agents)
```

```
    value_of_answers = [0,1,2,3,4]

    dd = make_aggregate_prb(coeff_val=np.array(value_of_answers))

    x = []
    y = []
    for key in dd:
        x.append(key)
        y.append(dd[key])

    HK1.init_op(x_distrib=x, y_distrib=y)

    # Find agents below the threshold and their initial score
    min_ = np.min(x)
    mean_= value_of_answers[2]*3
    max_ = np.max(x)

    threshold = mean_

    initial_below_thr = get_index_below_thr(HK1.opinions, thr=threshold)
    avg1 = dic_mean(initial_below_thr)
    labels_initial_below = [key for key in initial_below_thr]

    score0 = standardize_score(avg1, min_, max_, mean_)

    # Run
    HK1.run()

    # Get the data from the end
    op_dic = HK1.opinions
    new_below_thr = {key:op_dic[key] for key in labels_initial_below}

    avg1 = dic_mean(new_below_thr)
    score_fin = standardize_score(avg1, min_, max_, mean_)

    if not np.isnan(score_fin):
        initial_score.append(score0)
        final_score.append(score_fin)
        correlation_score.append(country_corr)
        dic_HK1_deff[country] = score_fin

beep()
```

[60]:
```
x = np.array(correlation_score)
y = np.array(final_score)
```

```
plt.scatter(-x,y)
plt.xlabel("Isolation")
plt.ylabel("Final posit of the skeptics")
plt.grid()

print(stt.spearmanr(-x,y))
```

```
SpearmanrResult(correlation=-0.348127991311206, pvalue=7.248846565886133e-30)
```

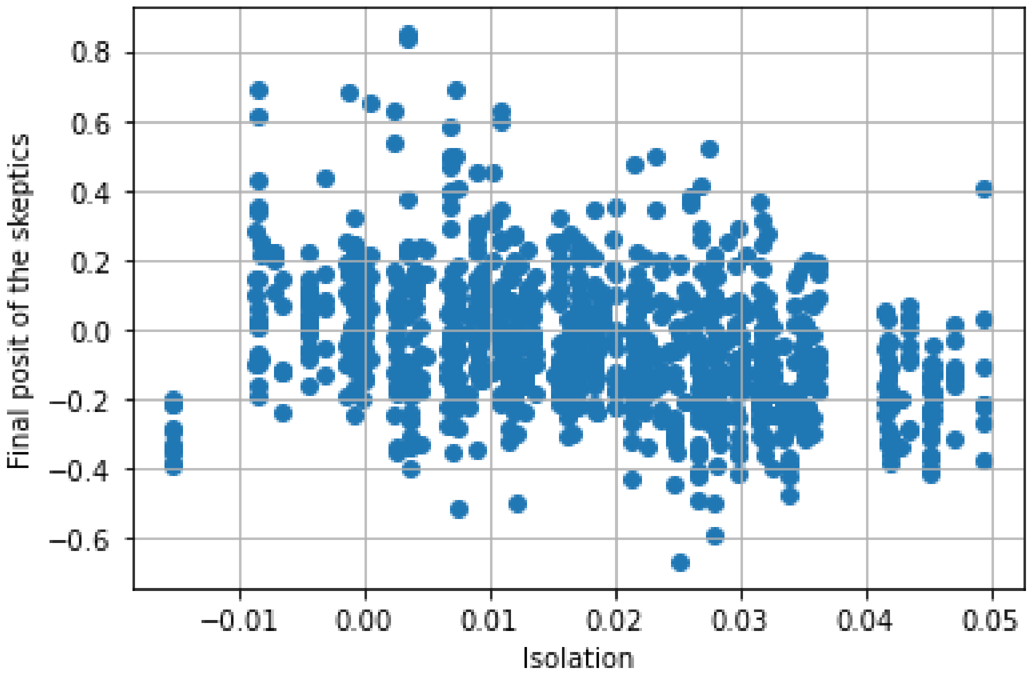

[ ]:

```
 
```

26.0.3 HK with geometric mean

[61]:

```
class HK(model):
    # initialization
    make_lab = generate_labels.ordered_numbers
    init_op = initialize_opinions.op_from_distrib

    # rules
    selec_rule = make_selection_rule.select_HK_random
    up_rule = make_interaction.geo_mean

    # observables
    mk_ob_op = make_observables.op_dic
```

```
    mk_ob_avg = make_observables.average_op
    mk_op_frq = make_observables.frequency_op

    # Run
    tick = ticks.tick_classic
    run = run.run_N_times
```

[62]:
```
HK1 = HK()
```

[63]:
```
    # parameters
N_agents = 1000
# Run_times = 0.1 # each agent will be run, on average this number of time!
HK1.parameters = {"eps":3, "N_agents":N_agents}
HK1.times_2_run = 100
```

[64]:
```
initial_score = []
final_score = []
correlation_score = []

repetitions = 1000

for rr in range(0,repetitions):

    # Select one random country
    country = random.choice(list(distributions_dic.keys()))
    country_corr = country_corr_dict[country]

    # Make the dictionary with the prb of answers
    dic_probability = distributions_dic[country] # this is the dictionary that␣
↪will be used for cheching the probabilities
    dic_probability = invert_dic(dic_probability)


    HK1.make_lab(N=N_agents)

    value_of_answers = [0,1,2,3,4]

    dd = make_aggregate_prb(coeff_val=np.array(value_of_answers))

    x = []
    y = []
    for key in dd:
        x.append(key)
        y.append(dd[key])

    HK1.init_op(x_distrib=x, y_distrib=y)
```

```
    # Find agents below the threshold and their initial score
    min_ = np.min(x)
    mean_= value_of_answers[2]*3
    max_ = np.max(x)

    threshold = mean_

    initial_below_thr = get_index_below_thr(HK1.opinions, thr=threshold)
    avg1 = dic_mean(initial_below_thr)
    labels_initial_below = [key for key in initial_below_thr]

    score0 = standardize_score(avg1, min_, max_, mean_)

    # Run
    HK1.run()

    # Get the data from the end
    op_dic = HK1.opinions
    new_below_thr = {key:op_dic[key] for key in labels_initial_below}

    avg1 = dic_mean(new_below_thr)
    score_fin = standardize_score(avg1, min_, max_, mean_)

    if not np.isnan(score_fin):
        initial_score.append(score0)
        final_score.append(score_fin)
        correlation_score.append(country_corr)

beep()
```

```
C:\Users\DeenoZord\anaconda3\lib\site-packages\scipy\stats\stats.py:410:
RuntimeWarning: divide by zero encountered in log
  log_a = np.log(a)
```

[65]:
```
x = np.array(correlation_score)
y = np.array(final_score)

plt.scatter(-x,y)
plt.xlabel("Isolation")
plt.ylabel("Final posit of the skeptics")
plt.grid()

print(stt.spearmanr(-x,y))
```

```
SpearmanrResult(correlation=-0.3834481054377014, pvalue=2.245937611833247e-36)
```

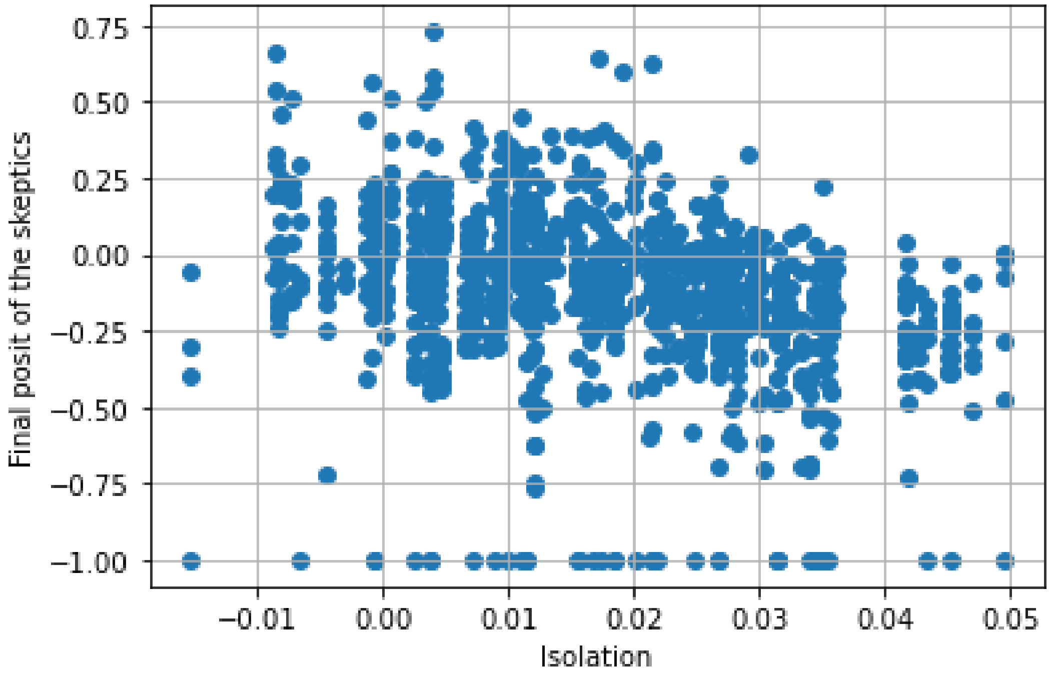


```
[ ]:
```

26.1 HK with H-mean

```
[66]: class HK(model):
          # initialization
          make_lab = generate_labels.ordered_numbers
          init_op = initialize_opinions.op_from_distrib

          # rules
          selec_rule = make_selection_rule.select_HK_random
          up_rule = make_interaction.h_mean

          # observables
          mk_ob_op = make_observables.op_dic
          mk_ob_avg = make_observables.average_op
          mk_op_frq = make_observables.frequency_op

          # Run
          tick = ticks.tick_classic
          run = run.run_N_times
```

```
[67]: HK1 = HK()
```

[68]:
```
    # parameters
N_agents = 1000
# Run_times = 0.1 # each agent will be run, on average this number of time!
HK1.parameters = {"eps":9, "N_agents":N_agents}
HK1.times_2_run = 100
```

[69]:
```
initial_score = []
final_score = []
correlation_score = []

repetitions = 1000

for rr in range(0,repetitions):

    # Select one random country
    country = random.choice(list(distributions_dic.keys()))
    country_corr = country_corr_dict[country]

    # Make the dictionary with the prb of answers
    dic_probability = distributions_dic[country] # this is the dictionary that␣
↪will be used for cheching the probabilities
    dic_probability = invert_dic(dic_probability)


    HK1.make_lab(N=N_agents)

    value_of_answers = [0,1,2,3,4]

    dd = make_aggregate_prb(coeff_val=np.array(value_of_answers))

    x = []
    y = []
    for key in dd:
        x.append(key)
        y.append(dd[key])

    HK1.init_op(x_distrib=x, y_distrib=y)

    # Find agents below the threshold and their initial score
    min_ = np.min(x)
    mean_= value_of_answers[2]*3
    max_ = np.max(x)

    threshold = mean_

    initial_below_thr = get_index_below_thr(HK1.opinions, thr=threshold)
    avg1 = dic_mean(initial_below_thr)
```

```
    labels_initial_below = [key for key in initial_below_thr]

    score0 = standardize_score(avg1, min_, max_, mean_)

    # Run
    HK1.run()

    # Get the data from the end
    op_dic = HK1.opinions
    new_below_thr = {key:op_dic[key] for key in labels_initial_below}

    avg1 = dic_mean(new_below_thr)
    score_fin = standardize_score(avg1, min_, max_, mean_)

    if not np.isnan(score_fin):
        initial_score.append(score0)
        final_score.append(score_fin)
        correlation_score.append(country_corr)

beep()
```

[70]:

```
x = np.array(correlation_score)
y = np.array(final_score)

plt.scatter(-x,y)
plt.xlabel("Isolation")
plt.ylabel("Final posit of the skeptics")
plt.grid()

print(stt.spearmanr(-x,y))
```

```
SpearmanrResult(correlation=-0.4309115832744135, pvalue=1.7843242263151594e-46)
```

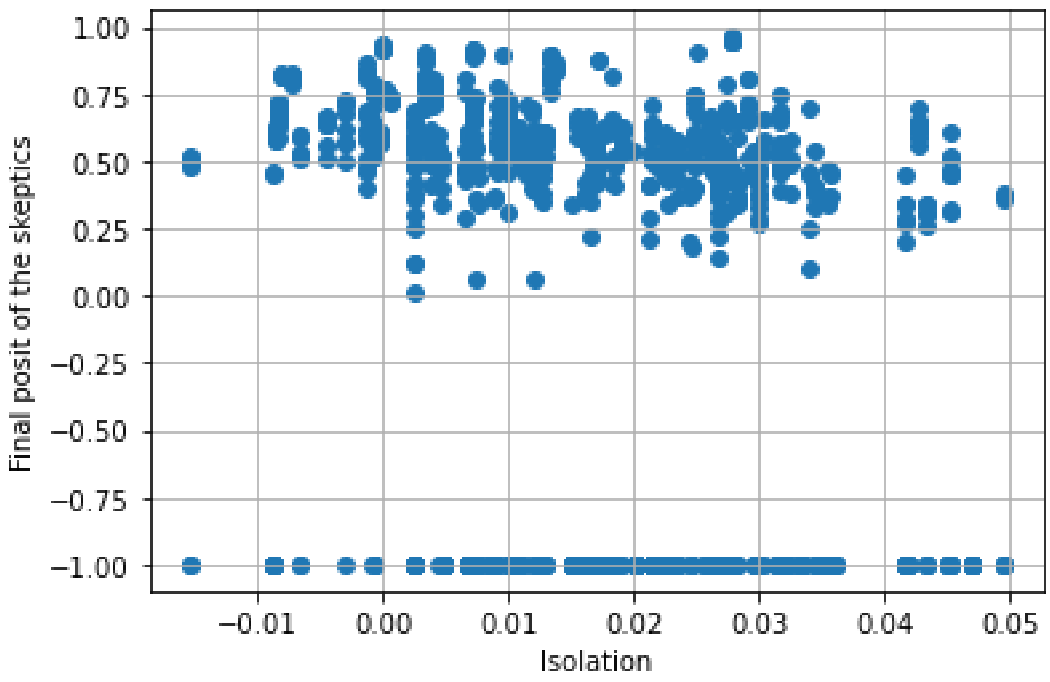


```
[ ]:
```

26.1.1 Axelrod model

```
[71]: class axelros_base(model):
          # initialization
          make_lab = generate_labels.ordered_numbers
          init_op = initialize_opinions.axel_from_distrib
      #     mk_network = mk_network.mk_lattice

          # rules
          selec_rule = make_selection_rule.select_random # (we are not using a lattice)
          up_rule = copy_trait_distance_

          # observables
      #     mk_observables = make_observables.average_op

          # Run
          tick = ticks.tick_classic
          run = run.run_N_times
```

```
[72]: # initialize
      repetitions = 1000
```

```
initial_score = []
final_score = []
correlation_score = []

USE_COUNTRIES = False

distance_exponent = 1

if USE_COUNTRIES:
    repetitions = len(countries)
else:
    repetitions = 1000

axel1 = axelros_base()

N_agents = 1000
axel1.times_2_run = 1000

value_of_answers = [0,1,2,3,4]
n_attitudes = 3

# Start the repetition
for rr in range(0,repetitions):

    # Select one random country
    if USE_COUNTRIES:
        country = countries[rr]
    else:
        country = random.choice(list(distributions_dic.keys()))

    country_corr = country_corr_dict[country]

    # Make the dictionary with the prb of answers
    dic_probability = distributions_dic[country] # this is the dictionary that␣
↪will be used for checking the probabilities
    dic_probability = invert_dic(dic_probability)


    # Define the max distance
    possible_answers = []
    prb_answers = []

    for key in dic_probability:
        t = transform_raw_vec(vec=key, coeff_val=value_of_answers)
        possible_answers.append(t)
        prb_answers.append(dic_probability[key])
```

```
    possible_answers = np.array(possible_answers)
    prb_answers = np.array(prb_answers)

    axel1.parameters = {"max_distance": (max(value_of_answers)-min(value_of_answers))*n_attitudes}

    # initialize the agents
    axel1.make_lab(N=N_agents)
    axel1.init_op(x_distrib=possible_answers, y_distrib=prb_answers)

    # Find agents below the threshold
    op_ = dic2array(axel1.opinions)[1]
    op2 = []
    for el in op_:
        op2.append(np.sum(el))
    op2 = np.array(op2)

    # parameters for normalization
    min_ = np.min(value_of_answers)*3
    mean_= value_of_answers[2]*3
    max_ = np.max(value_of_answers)*3

    index = np.array(range(0,len(op2)))
    initial_below_thr = index[np.array(op2)<=mean_]

    avg1_ = np.mean(op2[initial_below_thr])
    score0 = standardize_score(avg1_, min_, max_, mean_)

    initial_score.append(score0)

    # Run
    axel1.run()

    # Find agents below the threshold again
    op_ = dic2array(axel1.opinions)[1]
    op2 = []
    for el in op_:
        op2.append(np.sum(el))
    op2 = np.array(op2)

    avg1_ = np.mean(op2[initial_below_thr])
    score_fin = standardize_score(avg1_, min_, max_, mean_)

    if not np.isnan(score_fin):
        final_score.append(score_fin)
        correlation_score.append(country_corr)
```

[73]:
```
x = np.array(correlation_score)
y = np.array(final_score)

plt.scatter(-x,y)
plt.xlabel("Isolation")
plt.ylabel("Final posit of the skeptics")
plt.grid()

print(stt.spearmanr(-x,y))
```

```
SpearmanrResult(correlation=-0.5626052926425364, pvalue=1.5059615169704434e-84)
```

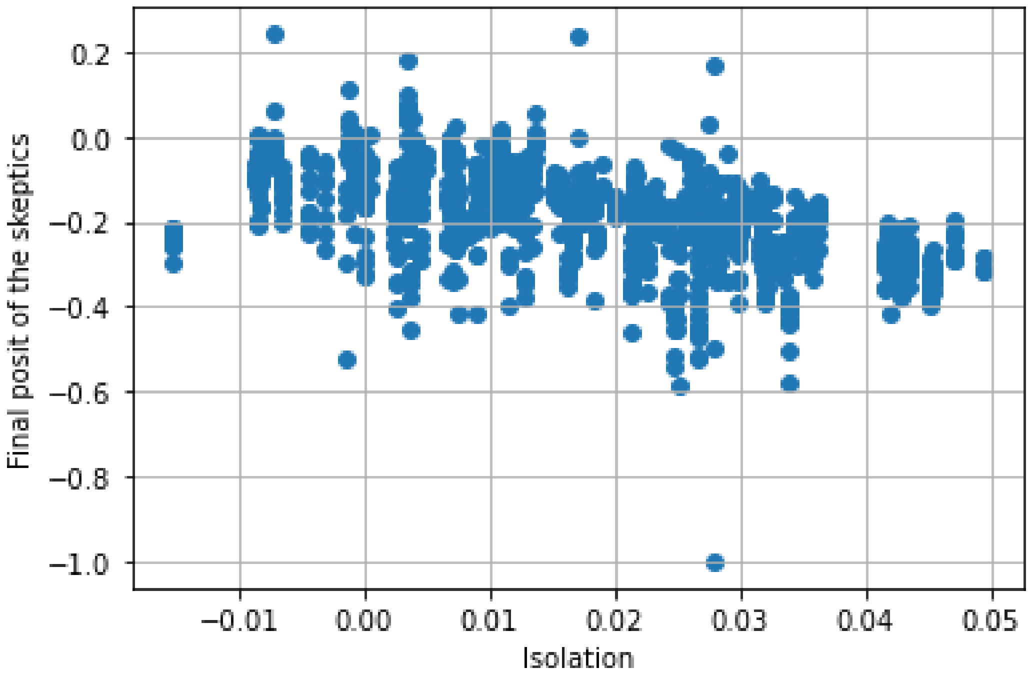

Part VI

Correlations and predictions

In this code we analyze how the isolation parameter correlates with the increase of anti-vaccination behavior

27 Import libraries

[1]:
```
# import packages
import sys
sys.path.insert(0, 'C:/Users/DeenoZord/Documents/All_Files_Laptop/Coding/
 ↪Pyton_Files/Functions_and_modules')

import numpy as np
import pandas as pd
import matplotlib.pyplot as plt
import importlib

from wgm2018_pack import *

import winsound
import scipy.stats as stt

from sklearn.linear_model import LinearRegression
from IPython.display import clear_output
```

28 List of attitudes

[2]:
```
vacc_neut_att = ['Vaccines important to children:Neither agree nor disagree',␣
 ↪'Vaccines Safe:Neither agree nor disagree', 'Vaccines Effective:Neither agree␣
 ↪nor disagree']

vacc_pos_att = ['Vaccines important to children:Strongly agree',
 'Vaccines important to children:Somewhat agree','Vaccines Safe:Strongly agree',
 'Vaccines Safe:Somewhat agree','Vaccines Effective:Strongly agree',
 'Vaccines Effective:Somewhat agree']
vacc_Wpos_att = ['Vaccines important to children:Somewhat agree',
                 'Vaccines Safe:Somewhat agree', 'Vaccines Effective:Somewhat␣
 ↪agree']
vacc_Spos_att = ['Vaccines important to children:Strongly agree',
                 'Vaccines Safe:Strongly agree', 'Vaccines Effective:Strongly␣
 ↪agree']
```

```
vacc_neg_att = ['Vaccines important to children:Somewhat disagree',
 'Vaccines important to children:Strongly disagree','Vaccines Safe:Somewhat␣
 ↪disagree',
 'Vaccines Safe:Strongly disagree','Vaccines Effective:Somewhat disagree',
 'Vaccines Effective:Strongly disagree']
vacc_Wneg_att = ['Vaccines important to children:Somewhat disagree',
                 'Vaccines Safe:Somewhat disagree','Vaccines Effective:Somewhat␣
 ↪disagree']
vacc_Sneg_att = ['Vaccines important to children:Strongly disagree',
                 'Vaccines Safe:Strongly disagree','Vaccines Effective:Strongly␣
 ↪disagree']
```

[3]:
```
dk_att = [
'Know Science:(DK)',
'Understand meaning Sci:(DK)',
'Study disease is science:(DK)',
'Poetry is science:(DK)',
'Learned Sci in Prim.School:(DK)',
'Learned Sci in Sec.School:(DK)',
'Learned Sci in College/Uni:(DK)',
'Searched Sci past 30d:(DK)',
'Searched Med past 30d:(DK)',
'Searched Sci:(DK)',
'Searched Med:(DK)',
'Confidence NGO:(DK)',
'Confidence Hospitals:(DK)',
'Trust neighborhood:(DK)',
'Trust government:(DK)',
'Trust Scientists:(DK)',
'Trust Journalists:(DK)',
'Trust Doctors:(DK)',
'Trust NGO workers:(DK)',
'Trust traditional Healers:(DK)',
'Trust science:(DK)',
'Trust Scientists 4 info:(DK)',
'Trust scientist intentions:(DK)',
'Trust scientists honesty:(DK)',
'Trust scientist in Med Comp intentions:(DK)',
'Trust scientists in Med Comp honesty:(DK)',
'Science benefits:(DK)',
'Science benefits you:(DK)',
'Science improve next gen:(DK)',
'Science will increase jobs:(DK)',
'Who trust most for Med Advice:(DK)',
'Trust gov 4 Med Advice:(DK)',
'Trust Doc 4 med advice:(DK)',
```

```
'Ever heard of vaccines:(DK)',
'Vaccines important to children:(DK)/(Refused)',
'Vaccines Safe:(DK)/(Refused)',
'Vaccines Effective:(DK)/(Refused)',
'Have Children:(DK)',
'Your Child Received Vax:(DK)',
'Religion:(DK)/(Refused)',
'Science disagreed w your religion:(DK)',
'(disagreement)Believe science or religion:(DK)',
]
```

[4]:
```
full_trust_att = [
'Trust neighborhood:A lot',
'Trust government:A lot',
'Trust Scientists:A lot',
'Trust Journalists:A lot',
'Trust Doctors:A lot',
'Trust NGO workers:A lot',
'Trust science:A lot',
'Trust Scientists 4 info:A lot',
'Trust scientist intentions:A lot',
'Trust scientists honesty:A lot',
'Trust scientist in Med Comp intentions:A lot',
'Trust scientists in Med Comp honesty:A lot',
'Trust gov 4 Med Advice:A lot',
'Trust Doc 4 med advice:A lot',
'Confidence Hospitals:Yes',
'Science benefits you:Yes',
'Science improve next gen:Yes',
]
```

[5]:
```
medium_trus_att = [
'Trust neighborhood:Some',
'Trust government:Some',
'Trust Scientists:Some',
'Trust Journalists:Some',
'Trust Doctors:Some',
'Trust NGO workers:Some',
'Trust traditional Healers:Some',
'Trust science:Some',
'Trust Scientists 4 info:Some',
'Trust scientist intentions:Some',
'Trust scientists honesty:Some',
'Trust scientist in Med Comp intentions:Some',
'Trust scientists in Med Comp honesty:Some',
'Science benefits:Some',
'Trust gov 4 Med Advice:Some',
```

```
'Trust Doc 4 med advice:Some'
]
```

[6]:
```
medium_distrust_att = [
'Trust neighborhood:Not much',
'Trust government:Not much',
'Trust Scientists:Not much',
'Trust Journalists:Not much',
'Trust Doctors:Not much',
'Trust NGO workers:Not much',
'Trust traditional Healers:Not much',
'Trust science:Not much',
'Trust Scientists 4 info:Not much',
'Trust scientist intentions:Not much',
'Trust scientists honesty:Not much',
'Trust scientist in Med Comp intentions:Not much',
'Trust scientists in Med Comp honesty:Not much',
'Trust gov 4 Med Advice:Not much',
'Trust Doc 4 med advice:Not much',
]
```

[7]:
```
full_distrust_att = [
'Trust neighborhood:Not at all',
'Trust government:Not at all',
'Trust Scientists:Not at all',
'Trust Journalists:Not at all',
'Trust Doctors:Not at all',
'Trust NGO workers:Not at all',
'Trust traditional Healers:Not at all',
'Trust science:Not at all',
'Trust Scientists 4 info:Not at all',
'Trust scientist intentions:Not at all',
'Trust scientists honesty:Not at all',
'Trust scientist in Med Comp intentions:Not at all',
'Trust scientists in Med Comp honesty:Not at all',
'Trust gov 4 Med Advice:Not at all',
'Trust Doc 4 med advice:Not at all',
]
```

[8]:
```
religion_att = ['Religion:Named a specific religion']
```

[9]:
```
refused_att = [
'Know Science:(Refused)',
'Understand meaning Sci:(Refused)',
'Study disease is science:(Refused)',
'Poetry is science:(Refused)',
'Learned Sci in Prim.School:(Refused)',
```

```
'Learned Sci in Sec.School:(Refused)',
'Learned Sci in College/Uni:(Refused)',
'Searched Sci past 30d:(Refused)',
'Searched Med past 30d:(Refused)',
'Searched Sci:(Refused)',
'Searched Med:(Refused)',
'Confidence NGO:(Refused)',
'Confidence Hospitals:(Refused)',
'Trust neighborhood:(Refused)',
'Trust government:(Refused)',
'Trust Scientists:(Refused)',
'Trust Journalists:(Refused)',
'Trust Doctors:(Refused)',
'Trust NGO workers:(Refused)',
'Trust traditional Healers:(Refused)',
'Trust science:(Refused)',
'Trust Scientists 4 info:(Refused)',
'Trust scientist intentions:(Refused)',
'Trust scientists honesty:(Refused)',
'Trust scientist in Med Comp intentions:(Refused)',
'Trust scientists in Med Comp honesty:(Refused)',
'Science benefits:(Refused)',
'Science benefits you:(Refused)',
'Science improve next gen:(Refused)',
'Science will increase jobs:(Refused)',
'Who trust most for Med Advice:(Refused)',
'Trust gov 4 Med Advice:(Refused)',
'Trust Doc 4 med advice:(Refused)',
'Ever heard of vaccines:(Refused)',
'Vaccines important to children:(DK)/(Refused)',
'Vaccines Safe:(DK)/(Refused)',
'Vaccines Effective:(DK)/(Refused)',
'Have Children:(Refused)',
'Your Child Received Vax:(Refused)',
'Religion:(DK)/(Refused)',
'Science disagreed w your religion:(Refused)',
'(disagreement)Believe science or religion:(Refused)',
]
```

[10]:
```
list_aggr_vacc = ["vacc_neut_att", "vacc_Wpos_att", "vacc_Spos_att",␣
 ↪"vacc_Wneg_att", "vacc_Sneg_att"]
list_aggr_trust = ["full_trust_att", "medium_trus_att", "medium_distrust_att",␣
 ↪"full_distrust_att"]
list_aggr_ref = ["refused_att", "dk_att"]
list_aggr_relig = ["religion_att"]
```

[11]:
```
list_aggr_vacc2 = ['vacc_neut_att',
 'vacc_Wpos_att',
 'vacc_Wneg_att',
 'vacc_Sneg_att']

list_aggr_vacc_Spos = ['vacc_Spos_att']
```

29 Functions

Define the functions to be used later

[12]:
```
# Generates a bootstrapped correlation from data
def bootstrap_corr(x,y,N,f):
    l = len(x)
    c_vec = []

    for i in range(0,N):
        r = np.floor(np.random.rand(N)*l).astype(int)
        xi = x[r]
        yi = y[r]
#         xi = x
#         yi = y

        ct = f(xi,yi)

        c_vec.append(ct)

    c_vec = np.array(c_vec)

    return [c_vec, np.mean(c_vec), np.std(c_vec)]
```

[13]:
```
def pearsonr_(x,y):
    [r,p] = stt.pearsonr(x,y)
    return r
```

[14]:
```
def spearmanr_positiv(*args):
    # this version returns only r, which will be used for making the network
    [r,p] = stt.spearmanr(*args)
    if r < 0:
        r = 0

    return r
```

[15]:

```
def make_graph_(list_of_nodes, metrics, exclude_same_question=True,␣
↪print_=False,  df=wgm_bool):
    G = nx.Graph()
    for i, node_i in enumerate(list_of_nodes):
        for j, node_j in enumerate(list_of_nodes):

            if print_:
                n_tot = len(list_of_nodes)**2
                current = (i*len(list_of_nodes))+(j)
                print(current,"/",n_tot, " = ", np.round(current/
↪n_tot,decimals=2))

            if j <= i:
                continue

            if exclude_same_question:
                if node_i.split(sep=':')[0] == node_j.split(sep=':')[0]:
                    # if they belong to the same question
                    continue

            [c1,c2] = get_col_values([node_i,node_j], df=df)
            weight = metrics(c1,c2)
            G.add_weighted_edges_from([(node_i,node_j,weight)])
    return G
```

[16]:
```
def p_corr_(x,y,z,corr_fun):
    # correlatuon between x and y after removing z

    # Need to reshape z as column for being accepted in LinearRegression
    zz = []
    for el in z:
        zz.append([el])
    z = np.array(zz)

    # Get the residuals on x
    reg = LinearRegression().fit(z, x)
    x_predicted = reg.predict(z)
    reg = LinearRegression().fit(z, x)
    x_residuals = x-x_predicted

    # Get the residuals on y
    reg = LinearRegression().fit(z, y)
    y_predicted = reg.predict(z)
    reg = LinearRegression().fit(z, y)
    y_residuals = y-y_predicted

    p_corr = corr_fun(x_residuals, y_residuals)
```

```
    return p_corr
```

```
[17]: def p_corr_multiple(x,y,z_s,corr_fun):
          # z_s is a list of elements to remove
          # correlatuon between x and y after removing z

          # Need to reshape z as column for being accepted in LinearRegression

          for z in z_s:
              zz = []
              for el in z:
                  zz.append([el])
              z = np.array(zz)

              # Get the residuals on x
              reg = LinearRegression().fit(z, x)
              x_predicted = reg.predict(z)
              reg = LinearRegression().fit(z, x)
              x_residuals = x-x_predicted

              # Get the residuals on y
              reg = LinearRegression().fit(z, y)
              y_predicted = reg.predict(z)
              reg = LinearRegression().fit(z, y)
              y_residuals = y-y_predicted

              x = x_residuals
              y = y_residuals

          p_corr = corr_fun(x_residuals, y_residuals)
          return p_corr
```

```
[18]: def check_avg_corr(list_of_aggregated_nodes, list_of_aggregated_nodes_n,␣
       ↪metrics, exclude_same_question=True, print_=False,  df=wgm_bool):
          # The first aggregated node is the one that you'll use to calculate the␣
       ↪correlation

      #     print(list_of_aggregated_nodes_n)
          a_node_i_name = list_of_aggregated_nodes_n[0]
          a_node_i = list_of_aggregated_nodes[0]

          count = 0
          for j, a_node_j_name in enumerate(list_of_aggregated_nodes_n):
              if j == 0:
                  pass
              else:
                  a_node_j = list_of_aggregated_nodes[j]
```

```
#         count = 0
        avg_weight = 0
#             print(a_node_i)
        for ii, node_i in enumerate(a_node_i):
            for jj, node_j in enumerate(a_node_j):

                if exclude_same_question:
                    if node_i.split(sep=':')[0] == node_j.split(sep=':')[0]:
                        # if they belong to the same question
                        continue

                [c1,c2] = get_col_values([node_i,node_j],df=df)
                weight = metrics(c1,c2)

                if not np.isnan(weight):
                    avg_weight = (weight + avg_weight*count)/(count+1)
                    count += 1

    return avg_weight
```

[ ]:

```

```

30 Import the datasets

Notice that the Wellcome Global Monitor has already been imported

30.0.1 Dataset on vaccination coverage

Source: https://apps.who.int/gho/data/node.main.A824?lang=en

Also available at: https://www.kaggle.com/lsind18/who-immunization-coverage

Note: when a future version is released, make sure to select the right column. Indeed, when also the 2020 data will be available, column 1 will not be anymore the 2019 data, but the 2020!

[19]:

```
df_vacc_coverage = pd.read_csv("MCV2_2019.csv")
df_vacc_coverage.head()
```

[19]:

```
    Unnamed: 0  \
0      Country
1  Afghanistan
2      Albania
3      Algeria
4      Andorra

  Measles-containing-vaccine second-dose (MCV2) immunization coverage by the
```

```
nationally recommended age (%)  \
0                                              2019
1                                                39
2                                                96
3                                                77
4                                                95

   Measles-containing-vaccine second-dose (MCV2) immunization coverage by the
nationally recommended age (%).1  \
0                                            2018.0
1                                              39.0
2                                              96.0
3                                              77.0
4                                              95.0

   Measles-containing-vaccine second-dose (MCV2) immunization coverage by the
nationally recommended age (%).2  \
0                                            2017.0
1                                              39.0
2                                              98.0
3                                              92.0
4                                              94.0

   Measles-containing-vaccine second-dose (MCV2) immunization coverage by the
nationally recommended age (%).3  \
0                                            2016.0
1                                              39.0
2                                              98.0
3                                              96.0
4                                              90.0

   Measles-containing-vaccine second-dose (MCV2) immunization coverage by the
nationally recommended age (%).4  \
0                                            2015.0
1                                              39.0
2                                              98.0
3                                              99.0
4                                              88.0

   Measles-containing-vaccine second-dose (MCV2) immunization coverage by the
nationally recommended age (%).5  \
0                                            2014.0
1                                              39.0
2                                              98.0
3                                              99.0
4                                              90.0
```

```
   Measles-containing-vaccine second-dose (MCV2) immunization coverage by the
nationally recommended age (%).6  \
0                                            2013.0
1                                              35.0
2                                              99.0
3                                              93.0
4                                              91.0

   Measles-containing-vaccine second-dose (MCV2) immunization coverage by the
nationally recommended age (%).7  \
0                                            2012.0
1                                              33.0
2                                              99.0
3                                              95.0
4                                              89.0

   Measles-containing-vaccine second-dose (MCV2) immunization coverage by the
nationally recommended age (%).8  \
0                                            2011.0
1                                              31.0
2                                              99.0
3                                              96.0
4                                              82.0

   ...  \
0  ...
1  ...
2  ...
3  ...
4  ...

   Measles-containing-vaccine second-dose (MCV2) immunization coverage by the
nationally recommended age (%).10  \
0                                            2009.0
1                                              24.0
2                                              98.0
3                                              98.0
4                                              82.0

   Measles-containing-vaccine second-dose (MCV2) immunization coverage by the
nationally recommended age (%).11  \
0                                            2008.0
1                                              23.0
2                                              98.0
3                                              96.0
4                                              86.0
```

```
   Measles-containing-vaccine second-dose (MCV2) immunization coverage by the
nationally recommended age (%).12  \
0                                            2007.0
1                                               4.0
2                                              95.0
3                                              95.0
4                                              80.0

   Measles-containing-vaccine second-dose (MCV2) immunization coverage by the
nationally recommended age (%).13  \
0                                            2006.0
1                                              18.0
2                                              94.0
3                                              98.0
4                                               NaN

   Measles-containing-vaccine second-dose (MCV2) immunization coverage by the
nationally recommended age (%).14  \
0                                            2005.0
1                                               9.0
2                                              97.0
3                                              94.0
4                                               NaN

   Measles-containing-vaccine second-dose (MCV2) immunization coverage by the
nationally recommended age (%).15  \
0                                            2004.0
1                                               2.0
2                                              96.0
3                                              94.0
4                                               NaN

   Measles-containing-vaccine second-dose (MCV2) immunization coverage by the
nationally recommended age (%).16  \
0                                            2003.0
1                                               NaN
2                                              93.0
3                                              96.0
4                                               NaN

   Measles-containing-vaccine second-dose (MCV2) immunization coverage by the
nationally recommended age (%).17  \
0                                            2002.0
1                                               NaN
2                                              93.0
3                                              93.0
4                                               NaN
```

```
   Measles-containing-vaccine second-dose (MCV2) immunization coverage by the
nationally recommended age (%).18  \
0                                            2001.0
1                                               NaN
2                                              90.0
3                                              73.0
4                                               NaN

   Measles-containing-vaccine second-dose (MCV2) immunization coverage by the
nationally recommended age (%).19
0                                            2000.0
1                                               NaN
2                                               NaN
3                                              78.0
4                                               NaN

[5 rows x 21 columns]
```

31 Dataset on attitudes towards vaccination

Source: https://www.thelancet.com/journals/lancet/article/PIIS0140-6736(20)31558-0/

[20]:
```python
df_trust = pd.read_excel("vaccine_trust.xlsx",sheet_name="raw_data")
df_trust.head()
```

[20]:
```
  country or territory who_region  count  sagree  sdisagree   question  \
0          Afghanistan        EMR   2080    1403         16  effective
1          Afghanistan        EMR    990     775          8  effective
2          Afghanistan        EMR   1724    1038         34  effective
3          Afghanistan        EMR   2080    1585          7  important
4          Afghanistan        EMR    990     838          5  important

          time  perc sagree  perc sdisagr
0  2015.833333    67.451923      0.769231
1  2018.538889    78.282828      0.808081
2  2019.915068    60.208817      1.972158
3  2015.833333    76.201923      0.336538
4  2018.538889    84.646465      0.505051
```

[ ]:

32 Test the correlations

32.0.1 Prediction in vaccination coverage

i.e. if the increase in vaccination coverage correlates with the isolation parameter

[21]:
```
year_col_base = 2 # this is for choosing the year [1 = 2019, 2 = 2018, etc...␣
 ↪range 2019-1980]
year_col_new = 1
# year_col_base will be 2018 and year_col_new will be 2019. This is for␣
 ↪calculating the increment in vaccination coverage

# Parameters for calculating the isolation parameter
metrics = pearsonr_
df = wgm_bool
exclude_same_question = True # Do not calculate corr between answers of the same␣
 ↪question

# Extra parameters
df_alt = df_vacc_coverage

# Initialize variables
aggr_wealth = dict()
dic_coverage_inc = dict()

list_of_aggregated_nodes_n = list_aggr_vacc_Spos+list_aggr_vacc2 # For␣
 ↪calculating the isolation

# Use the previous list to generate the variables
list_of_aggregated_nodes = []
for name in list_of_aggregated_nodes_n:
    exec("t = "+name)
    list_of_aggregated_nodes.append(t)

# inizialization of the lists

coverage_increase_vec = []
corr_score_vec = []
country_wealth = []
country_n_agree = []

# For each country in the df
country_list_df = [col for col in df.columns if "Country" in col] # select the␣
 ↪country columns
country_list_df = [col for col in country_list_df if np.sum(df[col]) > 0]  #␣
 ↪select the ones that have no 0 elements
```

```python
country_count = 0 # counter for checking how many countries we are analyzing
tot_country = len(country_list_df)

count_temp = 0
for count_col in country_list_df: # for each country
    country = count_col.split(sep=":")[1]

    country_count += 1
    if np.sum(df_alt["Unnamed: 0"] == country): # if the country is also in the other dataset
        count_temp += 1

        df_count = df [ df[count_col] == 1]

        # Get the vaccine rate for the different years
        mask_vacc = df_alt["Unnamed: 0"] == country # select the data from the specific country
        serie_base = df_alt.iloc[:,year_col_base] # 2018 data
        serie_new = df_alt.iloc[:,year_col_new] # 2019 data

        # Calculate the increase in vaccination coverage
        val_base = serie_base[mask_vacc].iloc[0]
        val_new = serie_new[mask_vacc].iloc[0]
        val = val_new-val_base
        val = val/val_base
        coverage_increase = val

        # Get the country wealth
        a = wgm_numeric [wgm_bool["Country:"+country]]
        wealth = a["Income Level"].unique()[0]

        # Get the isolation parameter
        avg_corr = check_avg_corr(list_of_aggregated_nodes, list_of_aggregated_nodes_n, metrics, exclude_same_question=exclude_same_question, print_=False,  df=df_count)

        # Store the data
        if (not np.isnan(coverage_increase)) and (not np.isnan(avg_corr)): # if the data of the country were not corrupted
            coverage_increase_vec.append(coverage_increase)
            corr_score_vec.append(avg_corr)
            country_wealth.append(wealth)

            # Get the percentage of totally agree
            a = wgm_bool [wgm_bool["Country:"+country]]
            n_agree = np.sum(a["Vaccines important to children:Strongly agree"])/len(a["Vaccines important to children:Strongly agree"])
```

```
            country_n_agree.append(n_agree)

            dic_coverage_inc[country] = coverage_increase

#             n_agree = np.sum(wgm_bool["Vaccines Effective:Strongly agree"])
#             n_agree = np.sum(wgm_bool["Vaccines Safe:Strongly agree"])
            clear_output()


coverage_increase_vec = np.array(coverage_increase_vec)
corr_score_vec = np.array(corr_score_vec)
country_n_agree = np.array(country_n_agree)

# make a df for the partial corr
df_pcorr = pd.DataFrame()
df_pcorr["vaccine cov"] = coverage_increase_vec
df_pcorr["corr scor"] = corr_score_vec
df_pcorr["wealth"] = country_wealth
df_pcorr["n_sagree"] = country_n_agree
```

[22]:
```
print("CORRELATION RESULTS:")
print("corr coverage increase & isolation = ", stt.
 ↪spearmanr(coverage_increase_vec,-corr_score_vec))
corr_fun = stt.spearmanr
print("partial corr (removing wealth) = ",␣
 ↪p_corr_(coverage_increase_vec,-corr_score_vec,country_wealth,corr_fun))
print("partial corr (n_agree) = ",␣
 ↪p_corr_(coverage_increase_vec,-corr_score_vec,country_n_agree,corr_fun))
print("partial corr removing both = ", p_corr_multiple(coverage_increase_vec,
                                                      -corr_score_vec,
                                                      ␣
 ↪[country_n_agree,country_wealth] ,corr_fun))
```

```
CORRELATION RESULTS:
corr coverage increase & isolation =
SpearmanrResult(correlation=-0.3096431228578482, pvalue=0.0010513472094203522)
partial corr (removing wealth) =
SpearmanrResult(correlation=-0.29082189056701163, pvalue=0.002155965916985571)
partial corr (n_agree) =  SpearmanrResult(correlation=-0.31119132236142766,
pvalue=0.0009889328639034539)
partial corr removing both =  SpearmanrResult(correlation=-0.2619463531969236,
pvalue=0.0059325139108974845)
```

Only the correlation between the two:

[23]:
```
print("Correlation betwee coverage increase & isolation")
[r,p] = stt.spearmanr(coverage_increase_vec,-corr_score_vec)
```

```
print("r = ", r)
print("p = ", p)
```

```
Correlation betwee coverage increase & isolation
r =  -0.3096431228578482
p =  0.0010513472094203522
```

Partial correlation after removing both wealth and trust:

[24]:
```
print("PARTIAL Correlation between coverage increase & isolation")
[r,p] =␣
 ↪p_corr_multiple(coverage_increase_vec,-corr_score_vec,[country_n_agree,country_wealth]␣
 ↪,corr_fun)
print("r = ", r)
print("p = ", p)
```

```
PARTIAL Correlation between coverage increase & isolation
r =  -0.2619463531969236
p =  0.0059325139108974845
```

Thus the higher the isolation, the stronger the decrease in vaccination rate

[ ]:
```

```

You can also calculate the correlation using bootstrapping

[25]:
```
[c,err]=bootstrap_corr(coverage_increase_vec,-corr_score_vec,100, pearsonr_)[1:]
print("Corr = ", c, " +- ", err)
```

```
Corr =  -0.32949208258180107  +-  0.06902317597248075
```

[ ]:
```

```

32.0.2 Test prediction in Trust

i.e. if the isolation parameter correlates with increase in distrust

Calculate the average correlations in each country

[26]:
```
df = wgm_bool

exclude_same_question = True # True or false
metrics = pearsonr_
list_of_aggregated_nodes_n = list_aggr_vacc_Spos+list_aggr_vacc2

# Turn the names into attitudes
list_of_aggregated_nodes = []
for name in list_of_aggregated_nodes_n:
    exec("t = "+name)
```

```
    list_of_aggregated_nodes.append(t)

no_vax_score_vec = []
corr_score_vec = []
n_notSpos = []
n_Spos = []

# For each country in the df
country_list_df = [col for col in df.columns if "Country:" in col] # select the␣
 ↪country columns
country_list_df = [col for col in country_list_df if np.sum(df[col]) > 0] #␣
 ↪select the ones that have no 0 elements

dic_distance = {}

for count_col in country_list_df:

    country_name = count_col.split(sep=':')[1]

    df_count = df [ df[count_col] == 1]

    # Get the isolation measur
    avg_corr = check_avg_corr(list_of_aggregated_nodes,␣
 ↪list_of_aggregated_nodes_n, metrics,␣
 ↪exclude_same_question=exclude_same_question, print_=False,  df=df_count)

    dic_distance[country_name] = avg_corr
    clear_output()
```

Calculate the increase in the level of distrust for each country

[27]:
```
dic_increase = {}
dic_trust_18 = {}

c_18 = []
c_19 = []
diff_vec = []

for country in df_trust["country or territory"].unique():

    c19_t = np.nan
    c18_t = np.nan

    df_t = df_trust [df_trust["country or territory"] == country]
    df_t = df_t [ df_t["question"] == "effective"]
```

```
    # Find the trust value for 2018 and 2019
    # notice that we may have multiple 2018 values for the same country,
    # in this case, we take the earliest one
    tt2018 = 3000
    for li in range(len(df_t.iloc[:,0])):
        line = df_t.iloc[li,:]

        tt2 = line["time"]

        if tt2>2018 and tt2<2020:

            if tt2>2019:
                c19_t = line["perc sdisagr"]

            if tt2<2019 and tt2018>tt2:
                tt2 = tt2
                c18_t = line["perc sdisagr"]


    if not (np.isnan(c19_t) or np.isnan(c18_t)): # if none is corrupted
        diff = c19_t-c18_t

        dic_increase[country] = diff

        c_18.append(c18_t)
        c_19.append(c19_t)
        diff_vec.append(diff)
        dic_trust_18[country] = c18_t
```

Use the same names in the two dictionaries

[28]:
```
dic_increase['United States'] = dic_increase['USA']
dic_increase['United Kingdom'] = dic_increase['UK']

del dic_increase['UK']
del dic_increase['USA']
```

Select only the countries which are common to the two datasets

[29]:
```
x = []
y = []

for country in dic_increase:
    if country in dic_distance:
        x.append(dic_increase[country])
        y.append(dic_distance[country])
```

```
x = -np.array(x)
y = np.array(y)
```

[30]:
```
r,p = stt.pearsonr(x,y)
print("r = ", r)
print("p = ", p)
```

```
r =  0.3175692633416603
p =  0.04883571042187488
```

Thus the higher the isolation parameter the stronger the distrust in a country

[ ]:
```

```

If you want to repeat it for bootstrapped correlation...

[31]:
```
[c,err]=bootstrap_corr(x,y,100,pearsonr_)[1:]
print("Corr = ", c, " +- ", err)
print("Notice that here the second value is NOT the p-value but the standard␣
↪deviation")
```

```
Corr =  0.3003042766202958  +-  0.0892659271488426
Notice that here the second value is NOT the p-value but the standard deviation
```

[ ]:
```

```

33 Test predictions from confidence

Before we showed that isolation can be used for measuring "dynamic effects" (i.e. what will happen in the future). Here we show that distrust has only a static and not dynamic effect

Correlation between distrust and its future value

[32]:
```
print("Correlation between distrust in 2018 and its increase in 2019")
r,p = stt.pearsonr(c_18,diff_vec)
print("r = ", r)
print("p = ", p)
```

```
Correlation between distrust in 2018 and its increase in 2019
r =  0.014380609709720549
p =  0.9307594563031496
```

Correlation between distrust and future increase in vaccination coverage

[33]:
```
x = []
y = []

for country in dic_coverage_inc:
    if country in dic_trust_18:
```

```
        x.append(dic_coverage_inc[country])
        y.append(dic_trust_18[country])

x = np.array(x)
y = np.array(y)
```

[34]:

```
print("Correlation between trust in 2018 and increase in vaccination coverage in␣
 ↪2019")
r,p = stt.pearsonr(x,y)
print("r = ", r)
print("p = ", p)
```

```
Correlation between trust in 2018 and increase in vaccination coverage in 2019
r =  -0.13943278609065618
p =  0.4706786135876143
```

As we can see both of them ar non-significant showing how distrust provides a static information.

Part VII

Simulating policies

In this notebook we simulate different policies

34 Import packages

[1]:
```
# import packages
import sys
sys.path.insert(0, 'C:/Users/DeenoZord/Documents/All_Files_Laptop/Coding/
↪Pyton_Files/Functions_and_modules')

import numpy as np
import pandas as pd
import matplotlib.pyplot as plt
import importlib

# import usefulFunctions as uf
# importlib.reload(uf)

from wgm2018_pack import *

import winsound
import scipy.stats as stt

from sklearn.linear_model import LinearRegression
```

35 Functions

Functions to be used later

[2]:
```
def pearsonr_(x,y):
    [r,p] = stt.pearsonr(x,y)
    return r
```

[3]:
```
def check_avg_corr(list_of_aggregated_nodes, list_of_aggregated_nodes_n,␣
↪metrics, exclude_same_question=True, print_=False,  df=wgm_bool):
    # The first aggregated node is the one that you'll use to calculate the␣
↪correlation

#     print(list_of_aggregated_nodes_n)
    a_node_i_name = list_of_aggregated_nodes_n[0]
    a_node_i = list_of_aggregated_nodes[0]
```

```
    count = 0
    for j, a_node_j_name in enumerate(list_of_aggregated_nodes_n):
        if j == 0:
            pass
        else:
            a_node_j = list_of_aggregated_nodes[j]

    #          count = 0
            avg_weight = 0
    #              print(a_node_i)
            for ii, node_i in enumerate(a_node_i):
                for jj, node_j in enumerate(a_node_j):

    #                          if print_:
    #                              n_tot = len(list_of_nodes)**2
    #                              current = (i*len(list_of_nodes))+(j)
    #                              print(current,"/",n_tot, " = ", np.
 ↪round(current/n_tot,decimals=2))

                    if exclude_same_question:
                        if node_i.split(sep=':')[0] == node_j.split(sep=':')[0]:
                            # if they belong to the same question
                            continue

                    [c1,c2] = get_col_values([node_i,node_j],df=df)
                    weight = metrics(c1,c2)

                    if not np.isnan(weight):
                        avg_weight = (weight + avg_weight*count)/(count+1)
                        count += 1

    return avg_weight
```

[4]:
```
def booleanize(df, list_of_questions):
    df_bool = pd.DataFrame()
    list_of_attitudes = []

    for quest in list_of_questions:
    #     values = sorted(df[quest].unique()) # list of unique values of the␣
 ↪dataset
        values = (df[quest].unique()) # list of unique values of the dataset
    #     min_ = min_dic[quest]
    #     max_ = max_dic[quest]

        for value in values:
            if type(value) == type('dsf'):
                name = str(quest)+":"+str(value)
```

```
                df_bool[name] = df[quest] == value
                list_of_attitudes.append(name)
            else:
                if np.isnan(value): # if it's a refused answer
                    name = str(quest)+":"+"Ref"
                    try:
                        df_bool[name] = df_bool[name] | df[quest] == value
                    except:
                        list_of_attitudes.append(name)
                        df_bool[name] = df[quest] == value
                else:
                    name = str(quest)+":"+str(value)
                    df_bool[name] = df[quest] == value
                    list_of_attitudes.append(name)
    return df_bool
```

36 Lists

[5]:
```
list_aggr_vacc2 = ['vacc_neut_att',
 'vacc_Wpos_att',
 'vacc_Wneg_att',
 'vacc_Sneg_att']

list_aggr_vacc_Spos = ['vacc_Spos_att']
```

[6]:
```
vacc_neut_att = ['Vaccines important to children:Neither agree nor disagree',␣
 ↪'Vaccines Safe:Neither agree nor disagree', 'Vaccines Effective:Neither agree␣
 ↪nor disagree']

vacc_pos_att = ['Vaccines important to children:Strongly agree',
 'Vaccines important to children:Somewhat agree','Vaccines Safe:Strongly agree',
 'Vaccines Safe:Somewhat agree','Vaccines Effective:Strongly agree',
 'Vaccines Effective:Somewhat agree']
vacc_Wpos_att = ['Vaccines important to children:Somewhat agree',
                 'Vaccines Safe:Somewhat agree', 'Vaccines Effective:Somewhat␣
 ↪agree']
vacc_Spos_att = ['Vaccines important to children:Strongly agree',
                 'Vaccines Safe:Strongly agree', 'Vaccines Effective:Strongly␣
 ↪agree']

vacc_neg_att = ['Vaccines important to children:Somewhat disagree',
 'Vaccines important to children:Strongly disagree','Vaccines Safe:Somewhat␣
 ↪disagree',
 'Vaccines Safe:Strongly disagree','Vaccines Effective:Somewhat disagree',
 'Vaccines Effective:Strongly disagree']
vacc_Wneg_att = ['Vaccines important to children:Somewhat disagree',
```

```
                 'Vaccines Safe:Somewhat disagree','Vaccines Effective:Somewhat␣
 ↪disagree']
vacc_Sneg_att = ['Vaccines important to children:Strongly disagree',
                 'Vaccines Safe:Strongly disagree','Vaccines Effective:Strongly␣
 ↪disagree']
```

[7]:
```
vacc_neut_att2 = ['Vaccines important to children:3', 'Vaccines Safe:3',
                  'Vaccines Effective:3']
vacc_Wpos_att2 = ['Vaccines important to children:2',
                 'Vaccines Safe:2', 'Vaccines Effective:2']
vacc_Spos_att2 = ['Vaccines important to children:1',
                 'Vaccines Safe:1', 'Vaccines Effective:1']
vacc_Wneg_att2 = ['Vaccines important to children:4',
                 'Vaccines Safe:4','Vaccines Effective:4']
vacc_Sneg_att2 = ['Vaccines important to children:5',
                 'Vaccines Safe:5','Vaccines Effective:5']

list_of_aggregated_nodes_n = ['vacc_Spos_att2', 'vacc_neut_att2',␣
 ↪'vacc_Wpos_att2']

list_questions = ["Vaccines important to children", "Vaccines Safe","Vaccines␣
 ↪Effective"]
```

[ ]:
```

```

37 Calculate the initial isolation value

[8]:
```
df2_numeric = wgm_numeric.copy()
df2_numeric = df2_numeric[['Vaccines Safe', 'Vaccines Effective', 'Vaccines␣
 ↪important to children']]
df=booleanize(df2_numeric, list_questions)

# Extra parameters
exclude_same_question = True # True or false

metrics = pearsonr_

# Turn the names into attitudes
list_of_aggregated_nodes = []
for name in list_of_aggregated_nodes_n:
#     print("t = "+name)
    exec("t = "+name)
    list_of_aggregated_nodes.append(t)

# Get the isolation measur
```

```
avg_corr = check_avg_corr(list_of_aggregated_nodes, list_of_aggregated_nodes_n,␣
↪metrics, exclude_same_question=exclude_same_question, print_=False,  df=df)

base = avg_corr

print("Isolation without any intervention: ", -avg_corr)
```

```
Isolation without any intervention:  0.09322222093761008
```

[ ]:

```
```

38 Test policy

38.0.1 Bad method

[9]:

```
df_num = df2_numeric.copy()
koeff = 0.55 # the higher this value, the more people we'll randomly select for␣
↪the policy

count = 0
for col in df_num.columns: # for each column (i.e. for each attitude)
    line_current = df_num[col]
    line_1 = df_num[df_num.columns[0]]
    line_2 = df_num[df_num.columns[1]]
    line_3 = df_num[df_num.columns[2]]

    for i,el in enumerate(line_current): # for each person
        el1 = line_1[i]
        el2 = line_2[i]
        el3 = line_3[i]

        n_valid = np.sum([type(x)==int for x in [el1, el2, el3]]) # number of␣
↪valid answers

        if (n_valid == 3) and (el==2): # if all answers

            n_interesting1 = np.sum([x==1 for x in [el1, el2, el3]]) #

            if n_interesting1==2: # if it's already 5 it doesn't make sense
                if np.random.rand()<koeff:
                    count += 1
                    df_num[col][i]=1

# Booleanize
df3_bool = booleanize(df_num, list_questions)
```

```
# Calculate the distance

df = df3_bool

# Extra parameters
exclude_same_question = True # True or false

metrics = pearsonr_

# Turn the names into attitudes
list_of_aggregated_nodes = []
for name in list_of_aggregated_nodes_n:
#     print("t = "+name)
    exec("t = "+name)
    list_of_aggregated_nodes.append(t)

# Get the isolation measur
avg_corr = check_avg_corr(list_of_aggregated_nodes, list_of_aggregated_nodes_n,␣
 ↪metrics, exclude_same_question=exclude_same_question, print_=False,  df=df)

print("Isolation value after this intervention: ", -avg_corr)
print("Improvement due to intervention = ", (1-avg_corr/base))
print("People selected:",count/140000)
```

```
Isolation value after this intervention:  0.12210280440625056
Improvement due to intervention =  -0.3098036410006697
People selected: 0.04990714285714286
```

[ ]:

38.0.2 Intervention tailored at decreasing isolation

[10]:
```
## Make policy
df_num = df2_numeric.copy()
koeff = 0.299 # the higher this value, the more people we'll randomly select for␣
 ↪the policy

count = 0
for col in df_num.columns: # for each column (i.e. for each attitude)
    line_current = df_num[col]
    line_1 = df_num[df_num.columns[0]]
    line_2 = df_num[df_num.columns[1]]
    line_3 = df_num[df_num.columns[2]]

    for i,el in enumerate(line_current): # for each person
        el1 = line_1[i]
```

```
        el2 = line_2[i]
        el3 = line_3[i]

        n_valid = np.sum([type(x)==int for x in [el1, el2, el3]]) # number of␣
 ↪valid answers

        if (n_valid == 3) and (el<=2) and (el>1): # if all answers

            n_interesting1 = np.sum([x>1 for x in [el1, el2, el3]]) #

            if n_interesting1==3:
                if np.random.rand()<koeff:
                    count += 1
                    df_num[col][i]=df_num[col][i]-1

# Booleanize
df3_bool = booleanize(df_num, list_questions)

df = df3_bool

# Extra parameters
exclude_same_question = True # True or false

metrics = pearsonr_

# Turn the names into attitudes
list_of_aggregated_nodes = []
for name in list_of_aggregated_nodes_n:
#     print("t = "+name)
    exec("t = "+name)
    list_of_aggregated_nodes.append(t)

# Get the isolation measur
avg_corr = check_avg_corr(list_of_aggregated_nodes, list_of_aggregated_nodes_n,␣
 ↪metrics, exclude_same_question=exclude_same_question, print_=False,  df=df)

print("Isolation value after this intervention: ", -avg_corr)
print("Improvement due to intervention = ", (1-avg_corr/base))
print("People selected:",count/140000)
```

```
Isolation value after this intervention:  0.0444553188545165
Improvement due to intervention =  0.5231252977305842
People selected: 0.05076428571428571
```

[ ]:

```

```
